# Supplementary material for: Near-infrared light-triggered prodrug photolysis by one-step energy transfer
Source: Nat Commun. 2023 Dec 7;14:8112. doi: 10.1038/s41467-023-43805-y (PMC10703928; doi:10.1038/s41467-023-43805-y)
Supplement: Supplementary file 1 — Supplementary Information [file 41467_2023_43805_MOESM1_ESM.pdf]

Supplementary Information

Near-infrared Light-Triggered Prodrug Photolysis by One-step Energy Transfer

Kaiqi Long,[abc] Wen Lv,[cd] Zihan Wang,[abc] Yaming Zhang,[abc] Kang Chen,[ab] Ni Fan,[abc] Feiyang Li,[e] Yichi Zhang,[abc] Weiping Wang,[abc]*

a. State Key Laboratory of Pharmaceutical Biotechnology, The University of Hong Kong, Pokfulam, Hong Kong, China.

b. Department of Pharmacology & Pharmacy, Li Ka Shing Faculty of Medicine, The University of Hong Kong, Pokfulam, Hong Kong, China.

c. Laboratory of Molecular Engineering and Nanomedicine, Dr. Li Dak-Sum Research Centre, The University of Hong Kong, Pokfulam, Hong Kong, China

d. State Key Laboratory of Organic Electronics and Information Displays, Jiangsu Key Laboratory for Biosensors, Institute of Advanced Materials (IAM), School of Chemistry and Life Sciences, Nanjing University of Posts & Telecommunications, Nanjing, China.

e. School of Environmental and Chemical Engineering, Jiangsu University of Science and Technology, Zhenjiang, China.

Corresponding author: W. Wang, wangwp@hku.hk

Contents

1. Supplementary Methods 3

2. Supplementary Figures 11

3. Supplementary Tables..41

Supplementary References..43

1. Supplementary Methods

Synthesis of BODIPY photocage

Two BODIPY photocages, compounds **1** and **2** were used in this study (Supplementary Figure S1). BODIPY photocage **1** is commercially available (Sigma-Aldrich, Steinheim, Germany). BODIPY photocage **2** was obtained as the product of the iodination reaction of **1**, of which the detailed method was as follow.[1]

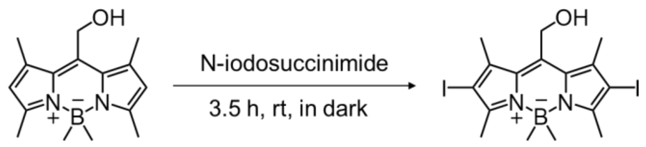

1 **2**

Supplementary Figure 1. Chemical structures of BODIPY photocage **1** and **2** and synthesis method from **1** to **2**.

BODIPY photocage **1** (41 mg, 0.15 mmol) was dissolved in dry tetrahydrofuran (2.5 mL). N-iodosuccinimide (74 mg, 0.33 mmol) was dissolved in dry tetrahydrofuran (1 mL) and was added dropwise into the flask. The solution was protected by N2 and kept in dark. After 3.5 h, the solvent was evaporated and the residue was purified by column chromatography (hexene/dichloromethane, 0% to 100% (v/v) in 20 min) on silica gel as a purple solid. Yield: 73 mg, 90.0%, ^{1}H NMR (500 MHz, $CDCl_3$): δ 5.63 (s, 2H), 2.56 (s, 6H), 2.51 (s, 6H), 0.21 (s, 6H). $[M+H]^+$: 522.98, found 523.00, 90% yield (71.3 mg).

Synthesis of [Os(bptpy)$_2$]· 2PF$_6$

The synthesis of [Os(bptpy)$_2$]·2PF$_6$ was conducted based on the reported method with modifications.[2]

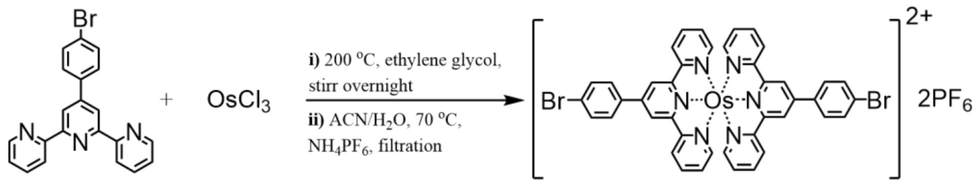

3

Supplementary Figure 2. Synthesis of [Os(bptpy)$_2$]·2PF$_6$ (compound 3).

Osmium (III) chloride hydrate (314.6 mg, 1 mmol) and 4'-(4-bromophenyl)-2,2':6',2''-terpyridine (766.5 mg, 2 mmol) were dissolved in 50 mL ethylene glycol and protected with nitrogen. The solution was heated to 200 ˚C and refluxed overnight under continuous stirring. The solution changed to dark purple after the reaction. The solution was dropwise added into excess water (500 mL) under vigorously stirring. The resulted purple precipitates were collected by filtration, washed by water and THF and dried. Then the resulted powder was dissolved in ACN (150 mL) and heated at 70 °C. Ammonium hexafluorophosphate (16.3 g, 10 mmol) was dissolved in water and added under stirring. After 2 h, the solution was cooled down, and the product was collected by filtration as dark purple precipitate and washed by water and ACN without further purification. ^{1}H NMR (500 MHz, DMSO-d6): δ (ppm), 9.52 (s, 4H), 9.09-9.10 (d, 4H), 8.38-8.40 (d, 4H), 7.99-8.01 (d, 4H), 7.92-7.95 (t, 4H), 7.43-7.44 (d, 4H), 7.20-7.23 (t, 4H). m/z = 968.03, found 968.00. Yield: 80% (1005.4 mg).

General procedures for the preparation of BODIPY ester prodrugs (4-9, 14)

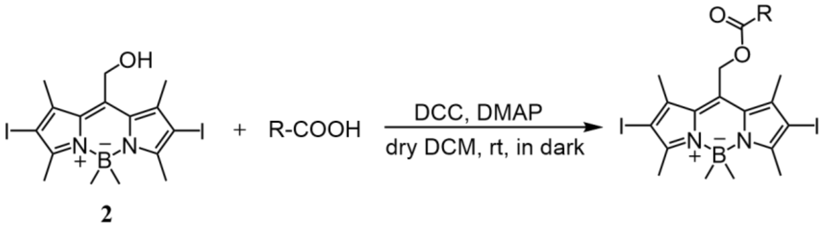

Supplementary Figure 3. Synthesis of BODIPY ester prodrugs.

7.5 mg BODIPY photocage **2** (0.014 mmol) was dissolved in 300 μL dry dichloromethane and protected in dark environment. Drug with carboxyl group (0.021 mmol), N, N'-dicyclohexylcarbodiimide (DCC) (6.9 mg, 0.0334 mmol), 4-dimethylaminopyridine (DMAP) (0.17 mg, 0.0014 mmol) were added to a sealable flask under the protection of nitrogen. Added 500 μL dry dichloromethane into the flask and stirred for 30 min, then injected the solution of **2** dropwise. The flask was placed in dark environment with tin foil covered on the surface. After 12 h, followed by the thin-layer chromatography to verify the complete reaction, the solvent was removed by evaporation. The residue was subjected to column chromatography on silica gel for purification (hexene/dichloromethane, 0% to 50% (v/v) in 30 min).

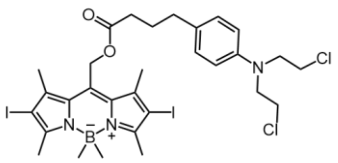

4 (BODIPY-Cb)

4: Purple-red solid. ^{1}H NMR (500 MHz, DMSO-d6): δ 6.96-6.98 (m, 2H), 6.62-6.64 (m, 2H) (aromatic CH), 5.34 (s, 2H), 3.69 (m, 8H) (CH_2), 3.31 (s, 6H), 2.36-2.45 (m, 9H), 1.76-1.79 (t,3H), 0.14 (s, 6H) (CH_3). $[M+H]^+$: 809.17, found 809.20. 89% yield (10.3 mg).

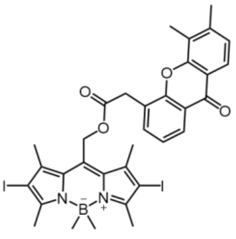

5 (BODIPY-DMXAA)

5: Dark-red solid. ^{1}H NMR (500 MHz, DMSO-d6): δ 8.19 (m, 2H), 7.87 (s, 1H), 7.68-7.70 (d, 1H), 7.48-7.50 (d, 1H) (aromatic CH), 4.71 (s, 2H), 3.00 (s, 2H) (CH_2), 2.34-2.43 (m, 9H), 1.20-1.21 (d, 9H), 0.16 (s, 6H) (CH_3). 92% yield (10.4 mg).

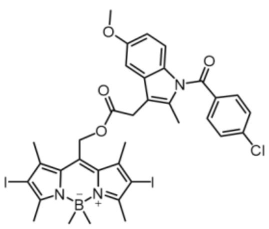

6 (BODIPY-IDM)

6: Purple-red solid. ^{1}H NMR (500 MHz, DMSO-d6): δ 8.17 (s, 2H), 7.47 (m, 5H) (aromatic CH), 6.79-6.85 (m, 2H), 5.20 (s, 2H) (CH_2), 3.79 (s, 3H), 3.49 (s, 3H), 2.04-2.09 (m, 12H), 1.71 (s, 3H), 1.09 (s, 3H) (CH_3). $[M+H]^+$: 862.75, found 862.70. 75% yield (9.3 mg).

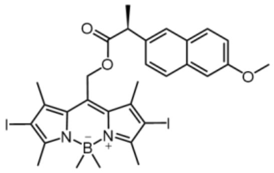

7 (BODIPY-NPX)

7: Purple-red solid. ^{1}H NMR (500 MHz, DMSO-d6): δ 7.63-7.67 (m, 2H), 7.59 (s, 1H), 7.21-7.25 (m, 2H), 7.04-7.06 (d, 1H) (aromatic CH), 5.27-5.31 (d, 1H), 5.13-5.16 (d, 1H) (CH_2), 3.96-3.98 (m, 1H) (CH), 3.77 (s, 3H), 3.23 (m, 6H), 2.35 (s, 6H), 2.03 (s, 6H), 1.38-1.40 (d, 3H) (CH_3). $[M+H]^+$: 735.22, found 735.20. 88% yield (9.3 mg).

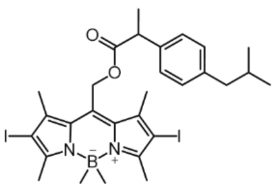

8 (BODIPY-IBF)

8: Red solid. ^{1}H NMR (500 MHz, DMSO-d6): δ 7.04-7.06 (D, 2H), 6.96-6.98 (d, 2H) (aromatic CH), 5.25 (d, 1H), 5.03 (d, 1H), 3.79 (d, 1H), 2.43 (m, 6H), 2.35 (m, 6H), 2.29-2.31 (d, 2H), 1.98

(m, 6H), 1.28-1.30 (d, 3H), 1.12 (s, 1H), 0.73-0.75 (d, 6H) (CH_3). $[M+H]^+$: 711.25, found 711.20. 88% yield (9.0 mg).

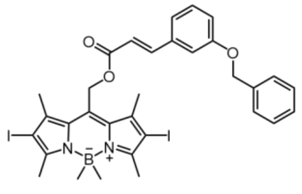

9 (BODIPY-BCA)

9: Purple-red solid. ^{1}H NMR (500 MHz, DMSO-d6): δ 7.52-7.57 (d, 2H), 7.15-7.30 (m, 8H) (aromatic CH), 6.90 (m, 1H), 6.64-6.68 (d, 1H), 5.28-5.30 (s, 2H), 4.96 (s, 2H), 2.33-2.35 (m, 12H), 2.22 (s, 6H) (CH_3). 65% yield (7.1 mg).

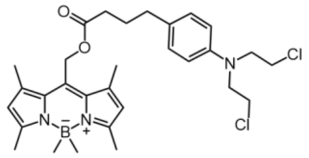

14 (BODIPY-Cb-2)

14: Orange solid. ^{1}H NMR (500 MHz, DMSO-d6): δ 6.96-6.98 (d, 2H), 6.62-6.65 (d, 2H), 6.29 (s, 2H) (aromatic CH), 5.29 (s, 2H), 3.68 (s, 10H), 3.31 (m, 4H) (CH_2), 2.43 (m, 9H), 2.34 (m, 6H), 1.75-1.79 (m, 3H) (CH_3). $[M+H]^+$: 557.38, found 557.40. 78% yield (6.2 mg).

General procedures for the preparation of BODIPY carbamate prodrugs (10-13)

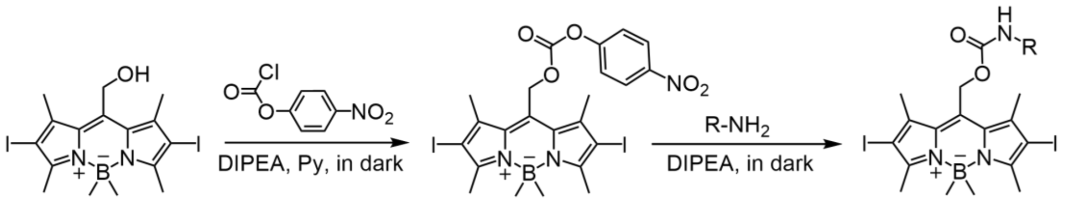

Supplementary Figure 4. Synthesis of BODIPY carbamate prodrugs.

BODIPY photocage **2** (50.0 mg, 0.096 mmol) was added to a 50 mL sealable flask. The flask was then vacuumed by pump and refilled by nitrogen gas. Dry dichloromethane (8 mL) was added to dissolve the solid under stirring. The flask was cooled to 0 ^{o}C in an ice-water bath. N, N-diisopropylethylamine (0.168 mL, 0.96 mmol) was added with a syringe with needle. Then p-nitrophenyl chloroformate (193.1mg, 0.96 mmol) was dissolved in dry dichloromethane (2 mL) and added into the above solution dropwise. Pyridine (31 μL, 0.38 mmol) was added with a syringe with needle. The flask was placed in dark environment with tin foil covered on the surface. After 12 h, followed by the thin-layer chromatography to verify the complete reaction, the solvent was removed by evaporation. The product (**15**, BODIPY-p-nitrobenzene) was purified by chromatography on a silica column (hexene/dichloromethane, 0% to 30% (v/v) in 30 min), followed by vacuum-drying for at least 24 h. Yield: 58% (38.3 mg).

BODIPY-p-nitrobenzene (**15**, 20 mg, 0.029 mmol) was dissolved with tetrahydrofuran (2 mL) in a 25 mL sealable flask, which was vacuumed by pump and refilled by nitrogen gas. Under stirring at room temperature, the drug with amino group (0.087 mmol) was dissolved in 0.5 mL DMF and added to the solution with a syringe with

needle. N, N-diisopropylethylamine (18 μL) was then added to the mixture. The flask was placed in dark environment with tin foil covered on the surface. After 4 h, the solvent was removed by evaporation and the product was collected by high-performance liquid chromatography (water/acetonitrile, 20% to 100% (v/v) in 60 min).

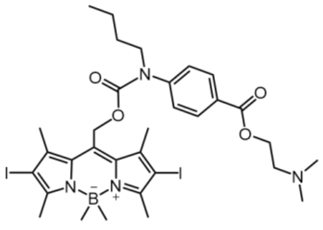

10 (BODIPY-TCI)

10: Dark-red solid. ^{1}H NMR (500 MHz, DMSO-d6): δ 7.64-7.66 (d, 2H), 6.52-6.54 (d, 2H) (aromatic CH), 4.71 (m, 2H), 4.26-4.28 (t, 2H), 2.99-3.03 (m, 2H), 2.84 (s, 2H) (CH_2), 2.49 (m, 6H), 2.37-2.40 (m, 12H) (CH_3), 1.45-1.79 (m, 2H), 1.29-1.34 (m, 2H) (CH_2), 0.84-0.87 (t, 3H), 0.04-0.07 (m ,6H) (CH_3). 26% yield (6.1 mg).

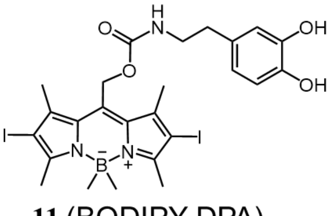

11 (BODIPY-DPA)

11: Dark-red solid. ^{1}H NMR (500 MHz, DMSO-d6): δ 8.63 (s, 1H), 8.55 (s, 1H) (OH), 7.36 (t, 1H) (NH), 6.49 (d, 1H), 6.43 (d, 1H), 6.29 (m, 1H) (aromatic CH), 5.11 (s, 2H), 3.97 (m, 4H) (CH_2), 2.38 (m, 6H), 2.25 (s, 9H), 1.10-1.14 (m, 3H) (CH_3). 43% yield (8.7 mg).

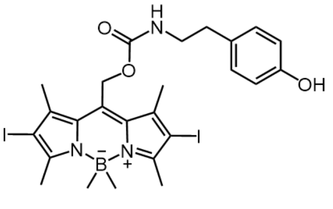

12 (BODIPY-TyrA)

12: Dark-red solid. ^{1}H NMR (500 MHz, DMSO-d6): δ 7.56-7.59 (m, 1H) (OH), 6.83-6.85 (d, 2H), 6.52-6.53 (d, 2H) (aromatic CH), 6.49 (d, 1H), 6.43 (d, 1H), 6.29 (m, 1H) (aromatic CH), 5.09 (s, 2H), 3.04-3.05 (m, 2H), 2.45-2.48 (m, 2H) (CH_2), 2.25 (s, 9H), 1.10 (s, 9H) (CH_3). 37% yield (7.3 mg).

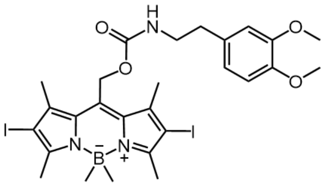

13 (BODIPY-HVA)

13: Dark-red solid. ^{1}H NMR (500 MHz, DMSO-d6): δ 7.50 (m, 1H) (NH), 6.80-6.83 (m, 2H), 6.69 (s, 1H) (aromatic CH), 5.22 (s, 2H), 2.84 (m, 2H), 2.63 (m, 2H) (CH_2), 2.36 (s, 9H), 1.23 (m, 6H), 0.66 (s, 3H) (aromatic CH), 0.12 (s, 6H). 50% yield (10.6 mg).

2. Supplementary Figures

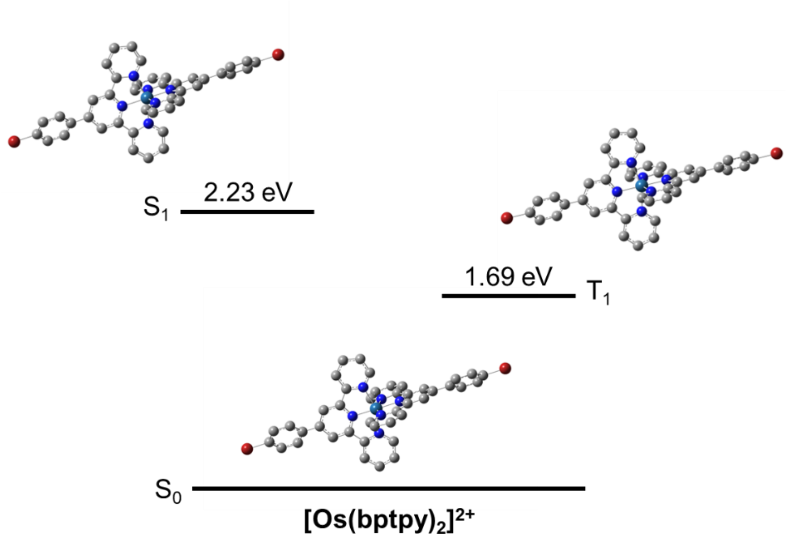

Supplementary Figure 5. Optimized excited state geometries and energy levels of $Os(bptpy)_2^{2+}$ determined at the B3LYP/6-31G(d) (LANL2DZ on Os) level with Gaussian 16C.01 software.

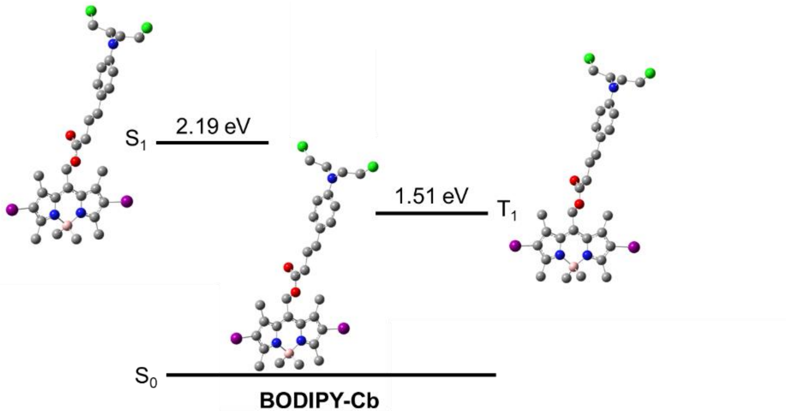

Supplementary Figure 6. Optimized excited state geometries and energy levels of BODIPY-Cb determined at the B3LYP/6-31G(d) (LANL2DZ on I) level with Gaussian 16C.01 software.

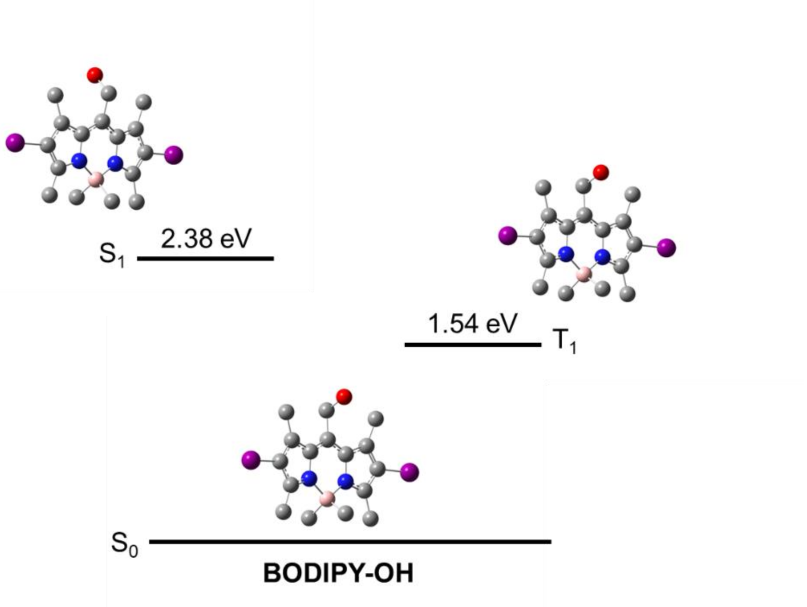

Supplementary Figure 7. Optimized excited state geometries and energy levels of BODIPY-OH determined at the B3LYP/6-31G(d) (LANL2DZ on I) level with Gaussian 16C.01 software.

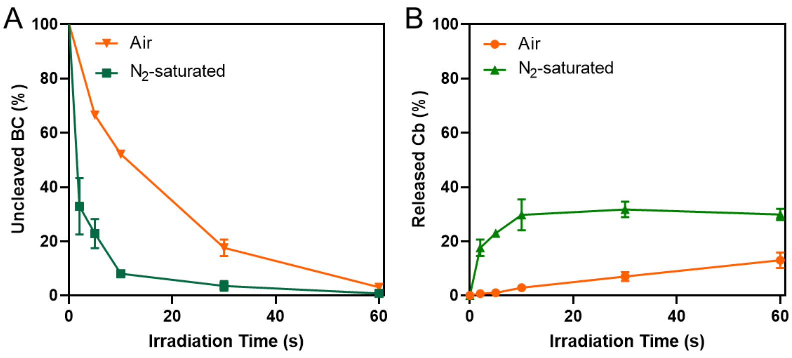

Supplementary Figure 8. (A) Photolysis rate of BODIPY-Cb upon 530 nm light irradiation for different time durations in air- or N_2-saturated solutions. (B) Generation rate of Cb upon 530 nm light irradiation for different time durations in air- or N_2-saturated solution. (BODIPY-Cb concentration: 10^{-3} M; Light irradiance: 50 mW/cm^2; n = 3 independent tests).

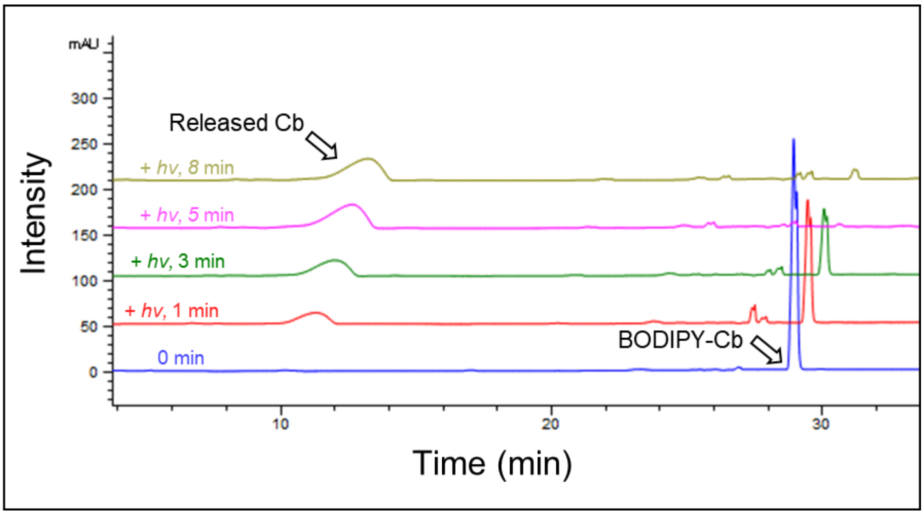

Supplementary Figure 9. HPLC elution curve of BODIPY-Cb with/without NIR light irradiation in the exist of 0.1 of $Os(bptpy)_2^{2+}$. (Prodrug concentration: 10^{-3} M; lamp parameter: 690 nm, 100 mW/cm^2, 30 min; time duration: 0-8 min; solution: 88% methanol, 10% dichloromethane, 2% acetone, N_2-saturated; λ_{abs} = 260 nm).

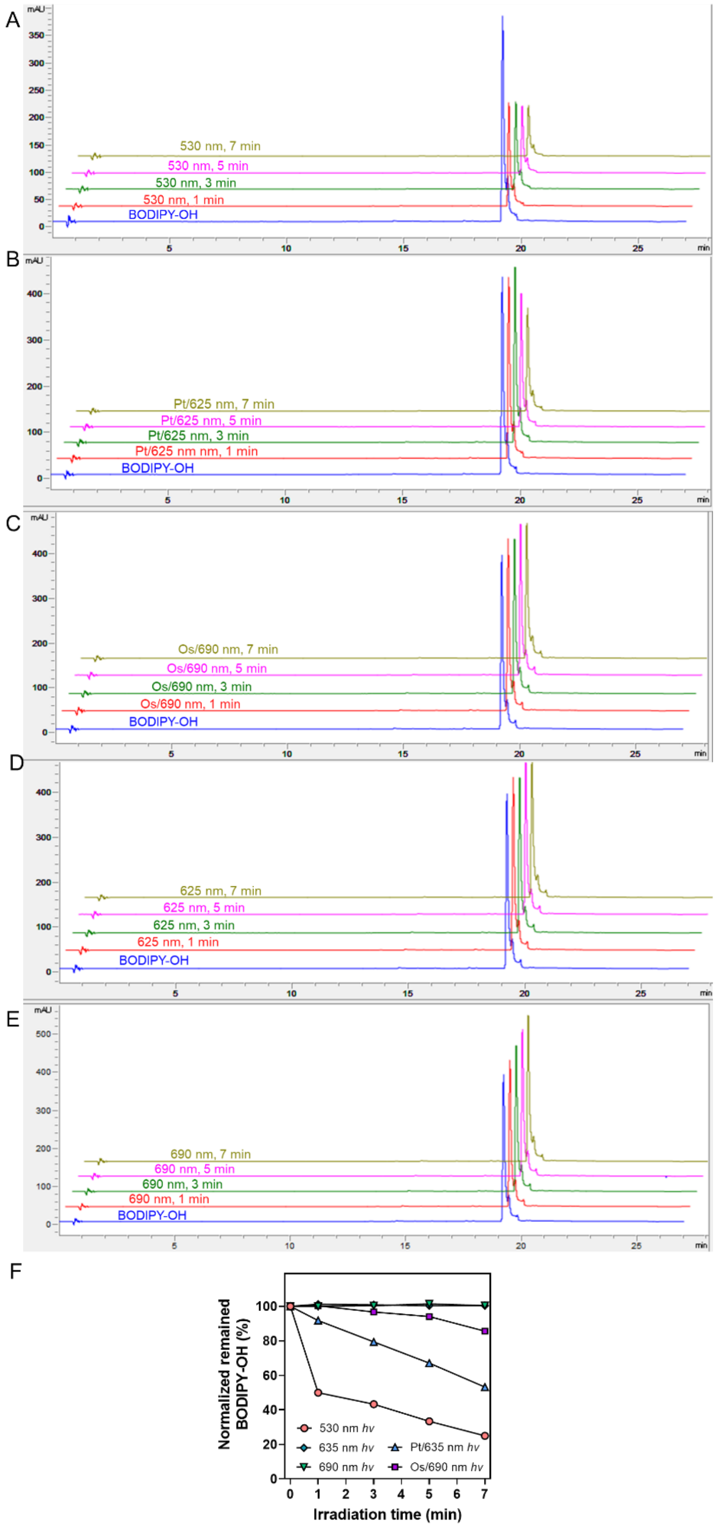

Supplementary Figure 10. (A-E) HPLC trace of BODIPY-OH photocage under 530 nm green light, 625 nm red light (with/without PtTPBP) and 690 nm NIR light (with/without $Os(bptpy)_2^{2+}$) (100 mW/cm^2, 0-7 min) (DAD detector, 540 nm). (F) Normalized remaining amount of BODIPY-OH in different groups.

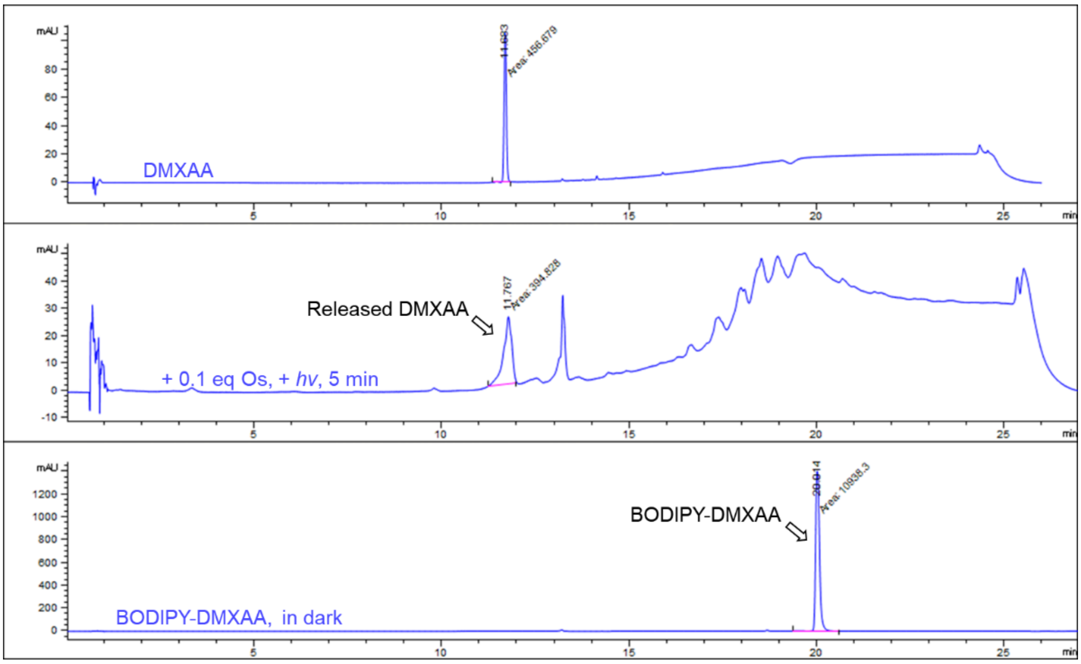

Supplementary Figure 11. HPLC elution curves of free DMXAA, BODIPY-DMXAA with/without NIR light irradiation in the existence of 0.1 eq Os(bptpy)$_2^{2+}$. (Prodrug concentration: 10^{-3} M; lamp parameter: 690 nm, 100 mW/cm^2, 30 min; time duration: 5 min; solution: 88% methanol, 10% dichloromethane, 2% acetone, N_2-saturated; λ_{abs} = 254 nm).

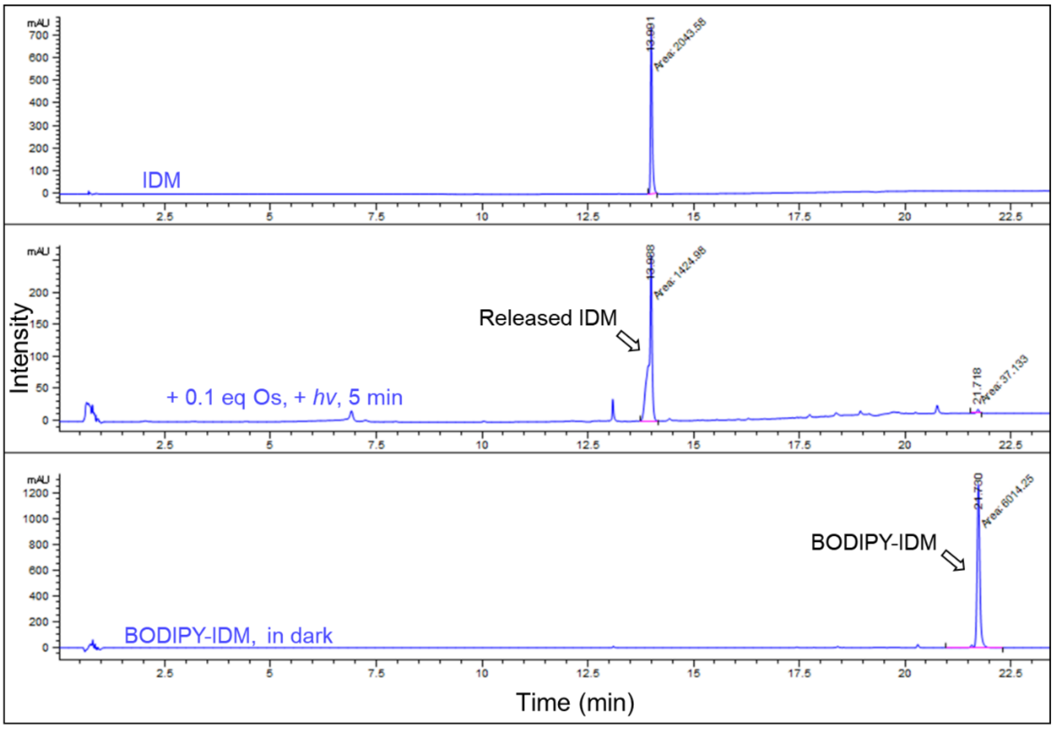

Supplementary Figure 12. HPLC elution curves of free IDM, BODIPY-IDM with/without NIR light irradiation in the existence of 0.1 eq $Os(bptpy)_2^{2+}$. (Prodrug concentration: 10^{-3} M; lamp parameter: 690 nm, 100 mW/cm^2, 30 min; time duration: 5 min; solution: 88% methanol, 10% dichloromethane, 2% acetone, N_2-saturated, λ_{abs} = 254 nm).

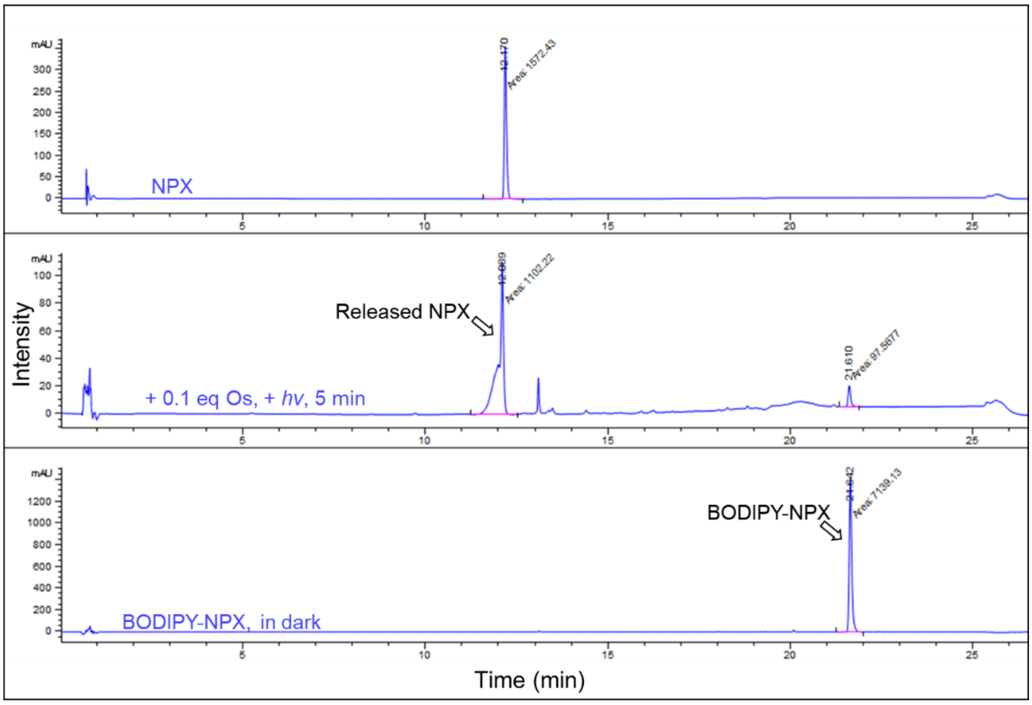

Supplementary Figure 13. HPLC elution curves of free NPX, BODIPY-NPX with/without NIR light irradiation in the existence of 0.1 eq Os(bptpy)$_2^{2+}$. (Prodrug concentration: 10^{-3} M; lamp parameter: 690 nm, 100 mW/cm^2, 30 min; time duration: 5 min; solution: 88% methanol, 10% dichloromethane, 2% acetone, N_2-saturated; λ_{abs} = 260 nm).

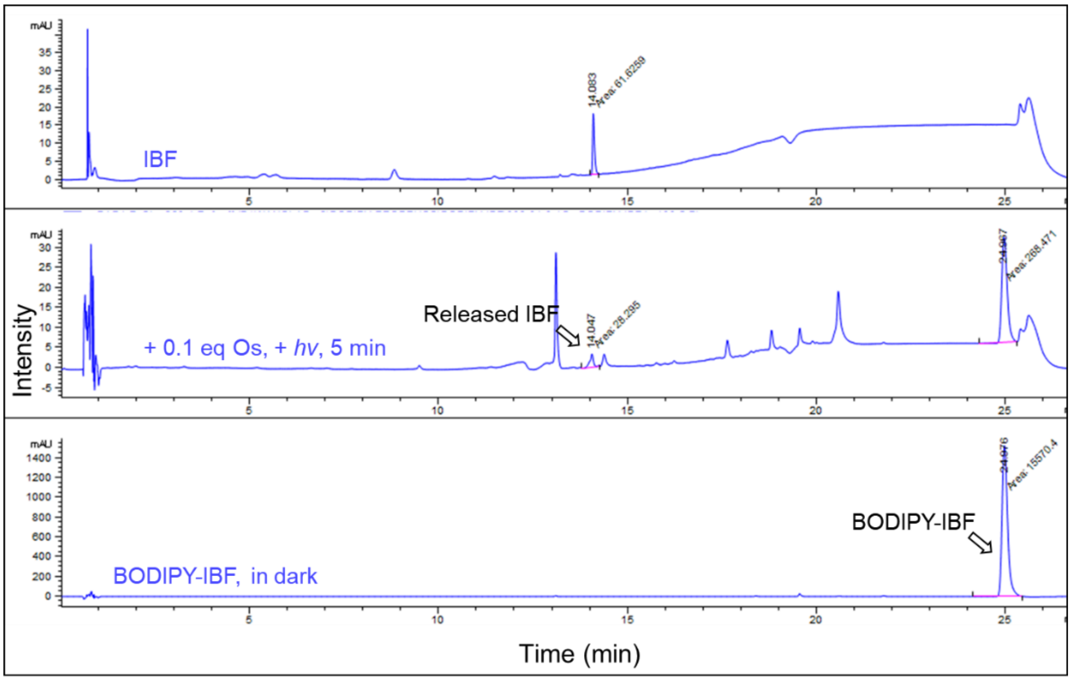

Supplementary Figure 14. HPLC elution curves of free IBF, BODIPY-IBF with/without NIR light irradiation in the existence of 0.1 eq $Os(bptpy)_2^{2+}$. (Prodrug concentration: 10^{-3} M; lamp parameter: 690 nm, 100 mW/cm^2, 30 min; time duration: 5 min; solution: 88% methanol, 10% dichloromethane, 2% acetone, N_2-saturated; λ_{abs} = 254 nm).

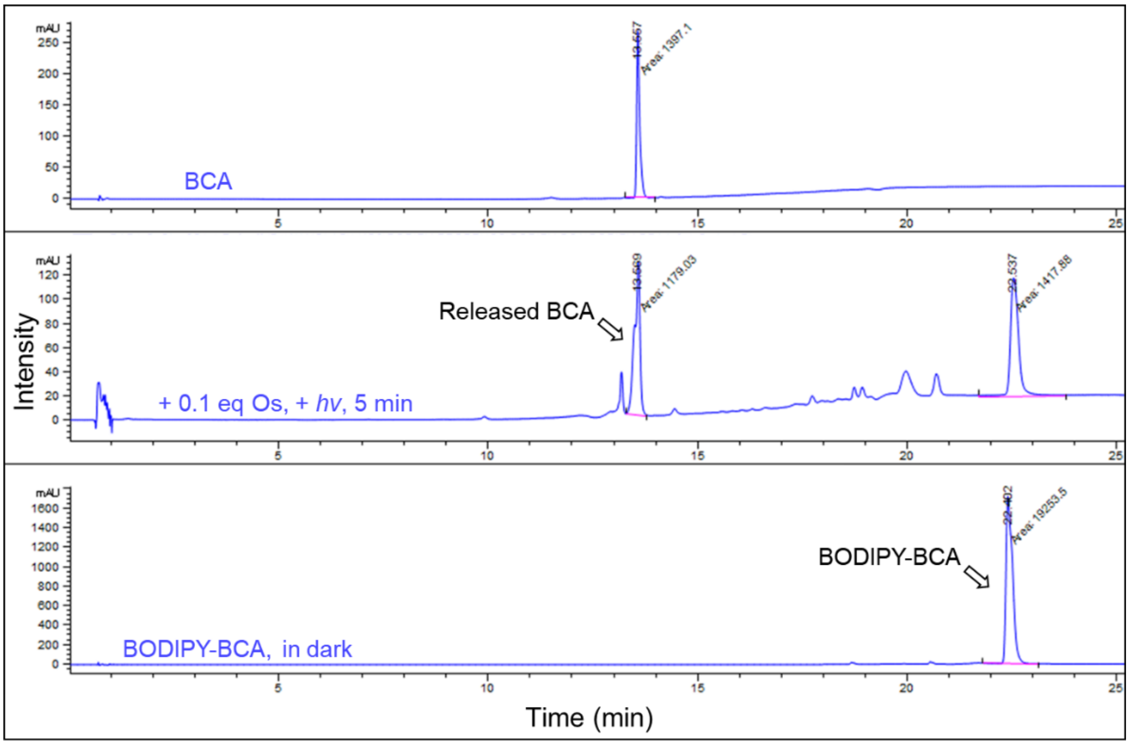

Supplementary Figure 15. HPLC elution curves of free BCA, BODIPY-BCA with/without NIR light irradiation in the existence of 0.1 eq $Os(bptpy)_2^{2+}$. (Prodrug concentration: 10^{-3} M; lamp parameter: 690 nm, 100 mW/cm^2, 30 min; time duration: 5 min; solution: 88% methanol, 10% dichloromethane, 2% acetone, N_2-saturated; λ_{abs} = 254 nm).

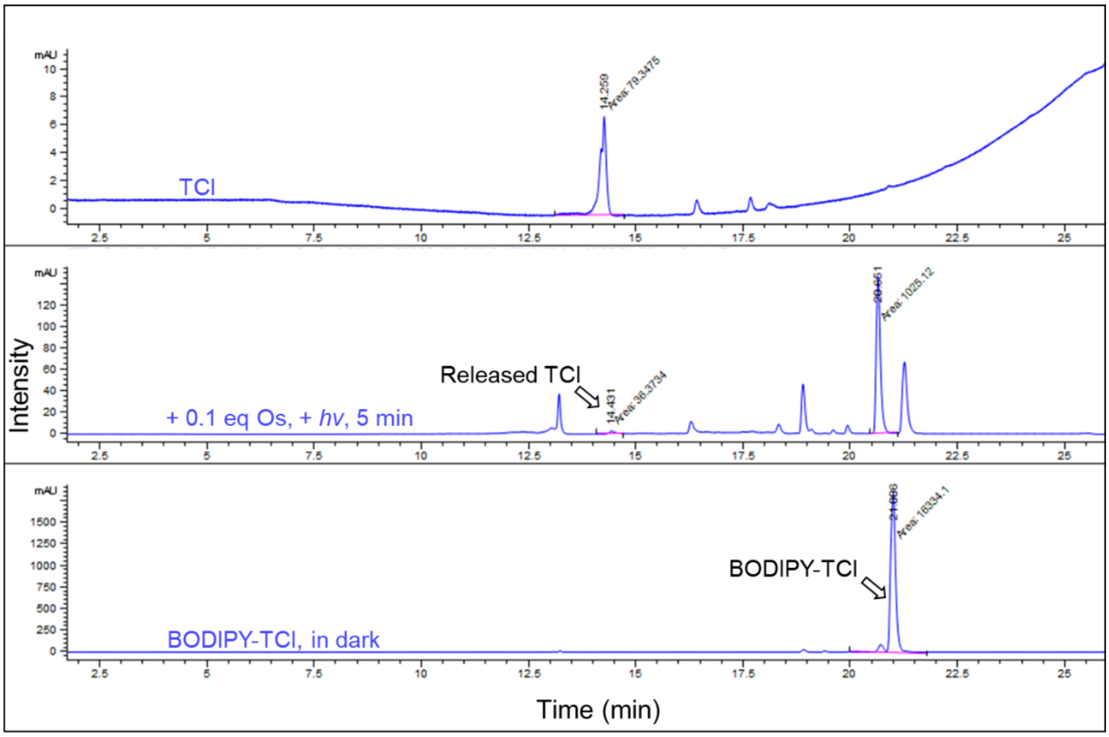

Supplementary Figure 16. HPLC elution curves of free TCI, BODIPY-TCI with/without NIR light irradiation in the existence of 0.1 eq $Os(bptpy)_2^{2+}$. (Prodrug concentration: 10^{-3} M; lamp parameter: 690 nm, 100 mW/cm^2, 30 min; time duration: 5 min; solution: 88% methanol, 10% dichloromethane, 2% acetone, N_2-saturated; λ_{abs} = 280 nm).

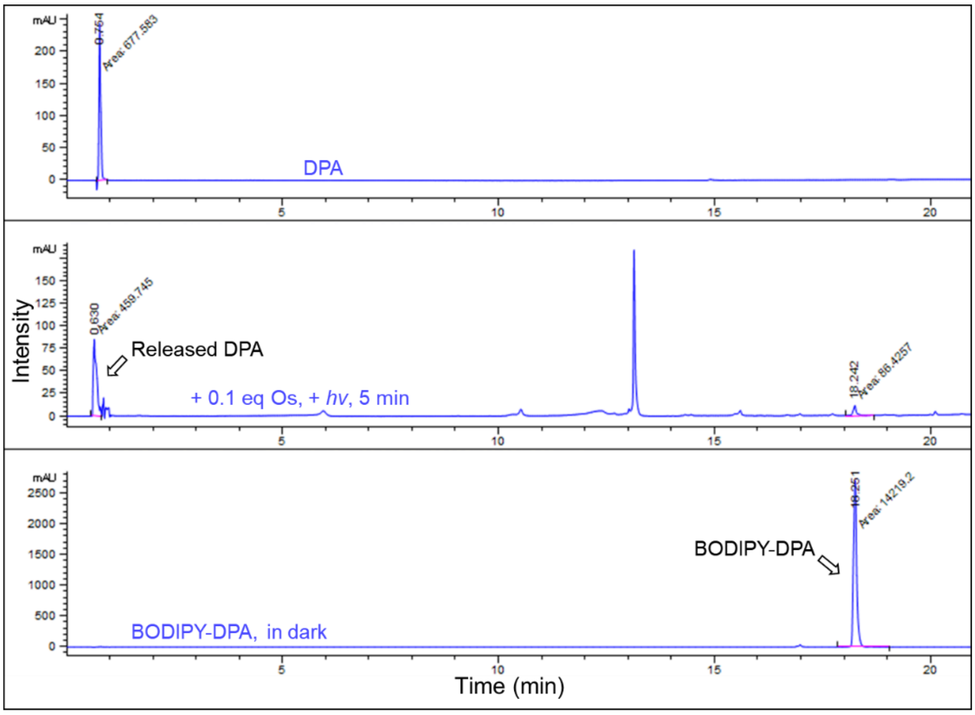

Supplementary Figure 17. HPLC elution curves of free DPA, BODIPY-DPA with/without NIR light irradiation in the existence of 0.1 eq Os(bptpy)$_2^{2+}$. (Prodrug concentration: 10^{-3} M; lamp parameter: 690 nm, 100 mW/cm^2, 30 min; time duration: 5 min; solution: 88% methanol, 10% dichloromethane, 2% acetone, N_2-saturated; λ_{abs} = 280 nm).

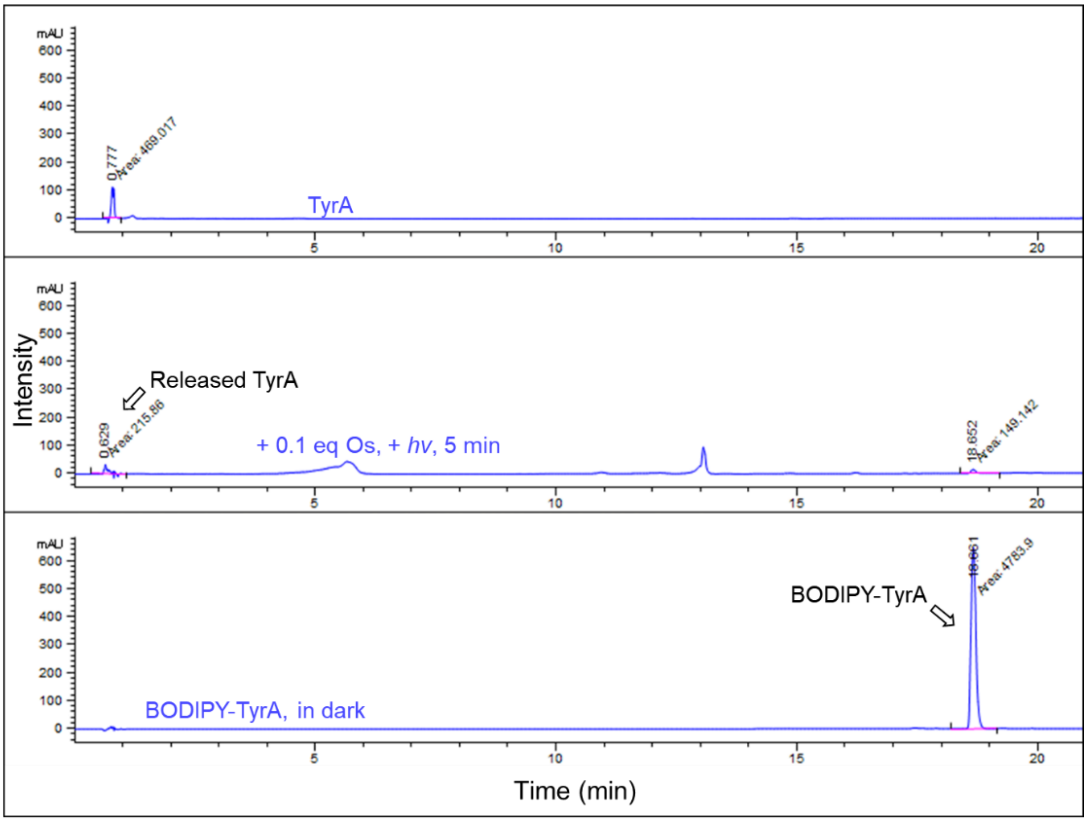

Supplementary Figure 18. HPLC elution curves of free TyrA, BODIPY-TyrA with/without NIR light irradiation in the existence of 0.1 eq Os(bptpy)$_2^{2+}$. (Prodrug concentration: 10^{-3} M; lamp parameter: 690 nm, 100 mW/cm^2, 30 min; time duration: 5 min; solution: 88% methanol, 10% dichloromethane, 2% acetone, N_2-saturated; λ_{abs} = 280 nm).

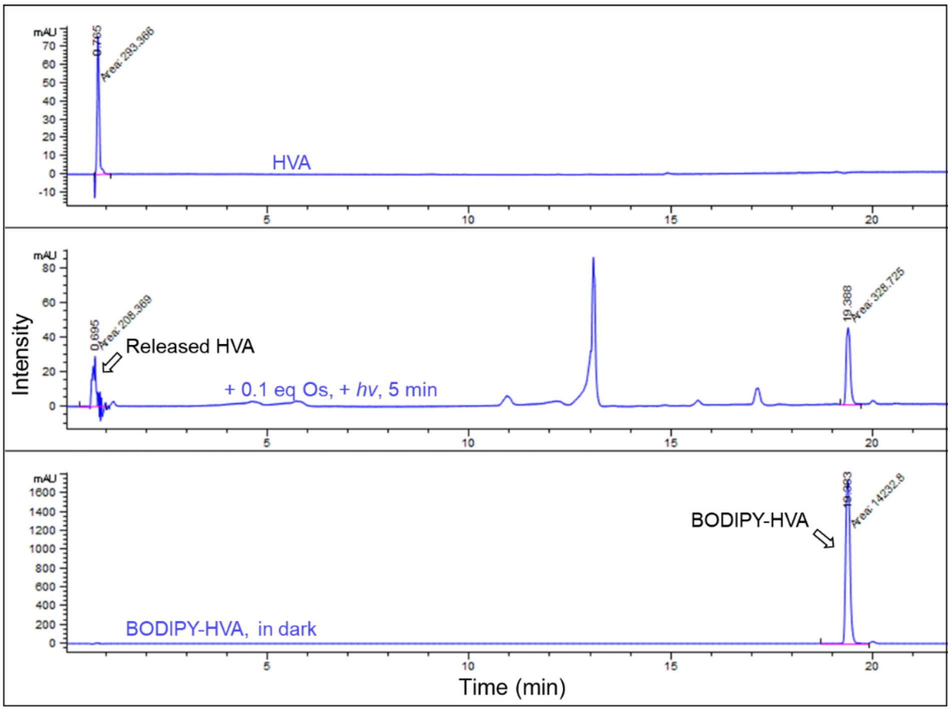

Supplementary Figure 19. HPLC elution curves of free HVA, BODIPY-HVA with/without NIR light irradiation in the existence of 0.1 eq $Os(bptpy)_2^{2+}$. (Prodrug concentration: 10^{-3} M; lamp parameter: 690 nm, 100 mW/cm^2, 30 min; time duration: 5 min; solution: 88% methanol, 10% dichloromethane, 2% acetone, N_2-saturated; λ_{abs} = 280 nm).

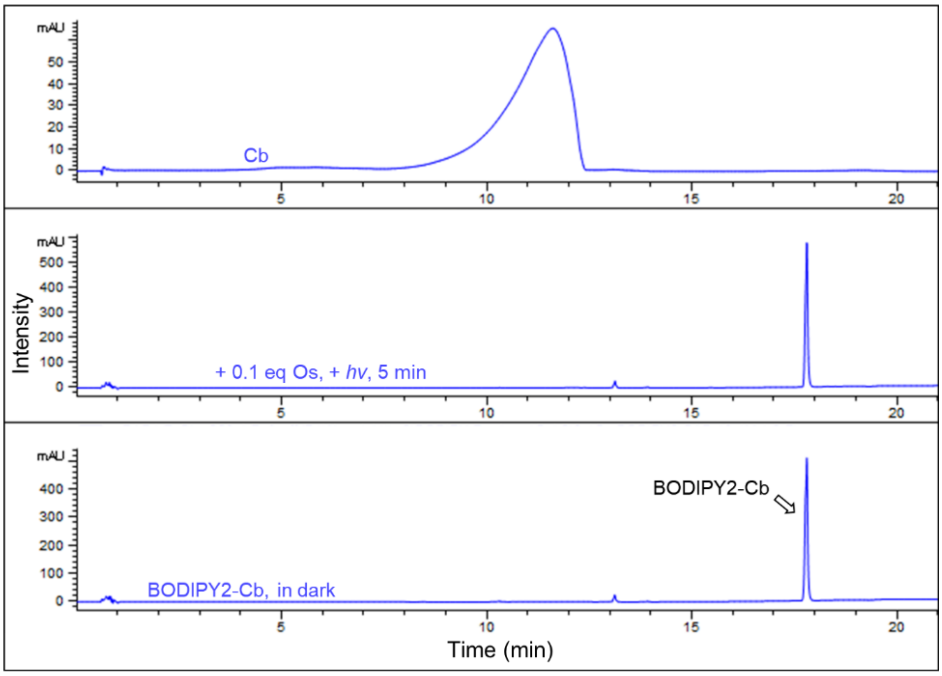

Supplementary Figure 20. HPLC elution curves of free Cb, BODIPY2-Cb with/without NIR light irradiation in the existence of 0.1 eq $Os(bptpy)_2^{2+}$. (Prodrug concentration: 10^{-3} M; lamp parameter: 690 nm, 100 mW/cm^2, 30 min; time duration: 5 min; solution: 88% methanol, 10% dichloromethane, 2% acetone, N_2-saturated; λ_{abs} = 260 nm).

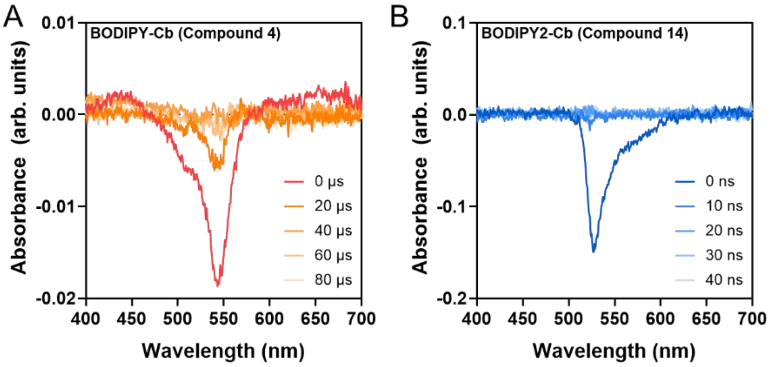

Supplementary Figure 21. Nanosecond transient absorption spectrum of (A) BODIPY-Cb and (B) BODIPY2-Cb in N_2-saturated toluene. (Concentration: 10^{-5} M; λ_{ex} = 355 nm).

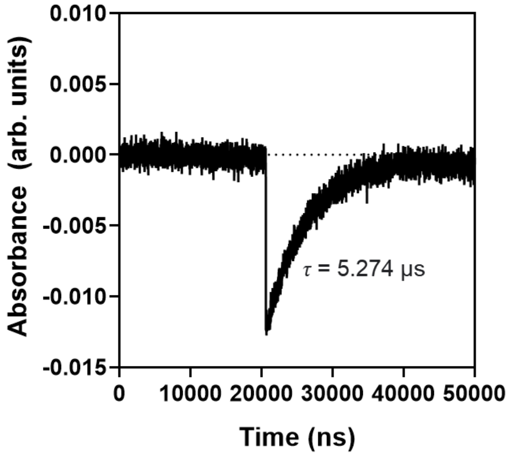

Supplementary Figure 22. Decay trace of BODIPY-Cb at 540 nm at 20 ^oC. (Concentration: 10^{-5} M; λ_{ex} = 355 nm).

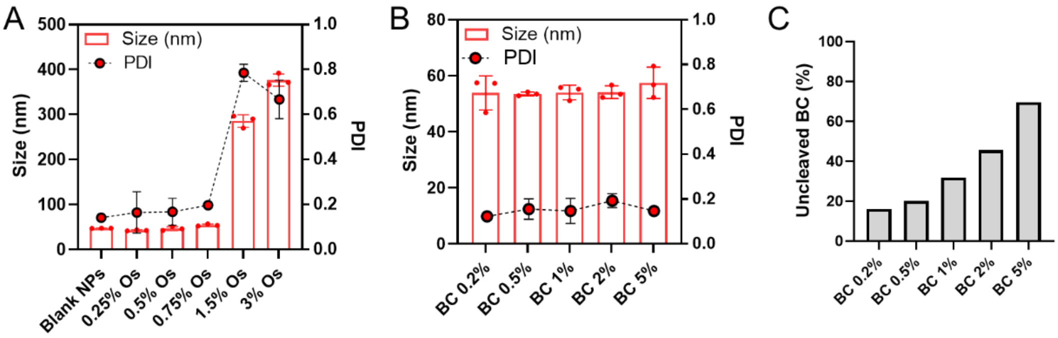

Supplementary Figure 23. Size, PDI of PLA-PEG NPs containing different contents of Os (A) and BC (B). Data were presented as mean ± SD, n = 3 independent experiments. (C) Uncleaved BC percentages of different Os/BC NPs (0.75% Os (w/w) with different feeding ratios of BC under NIR light (690 nm, 100 mW/cm^2, 30 min).

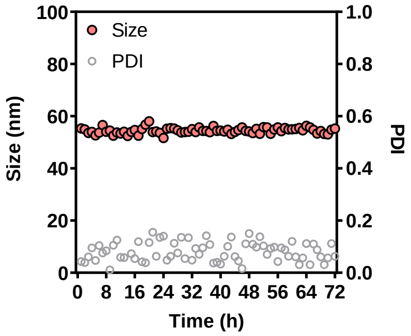

Supplementary Figure 24. Size and PDI of Os/BC NPs in PBS for 72 h under 37 °C.

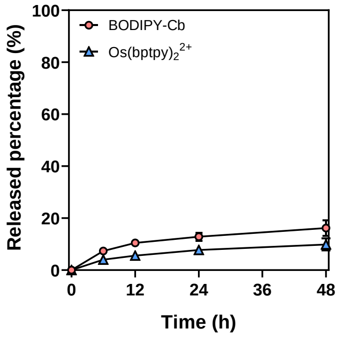

Supplementary Figure 25. Release profiles of BC and Os in nanoparticles in 37 °C within 48 h. Data were presented as mean ± SD, n = 3 independent experiments.

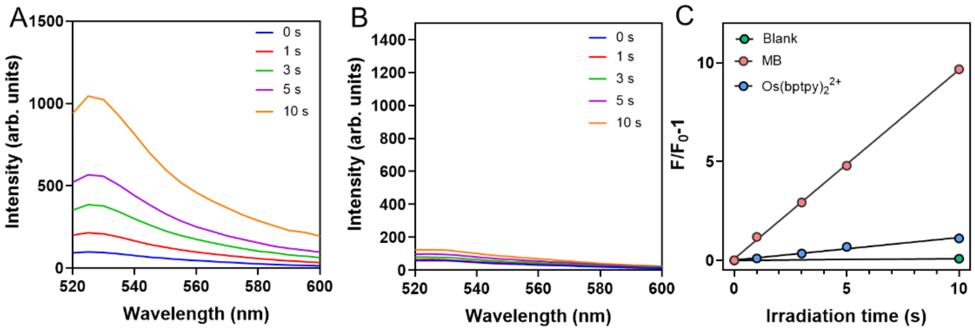

Supplementary Figure 26. Singlet oxygen generation of methyl blue (MB) (A) and Os NPs (B), detected by SOSG assay. (C) Time-dependent F/F_0-1 values at 525 nm under NIR light irradiation. (light irradiation: 690 nm, 10 mW/cm^2, 0-10 s)

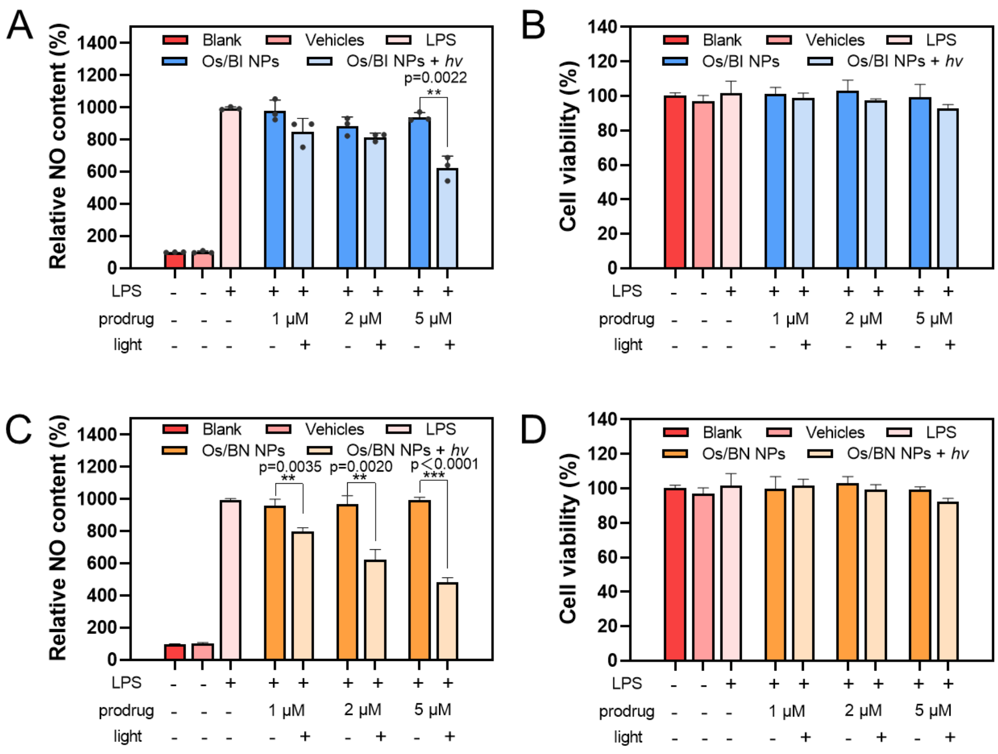

Supplementary Figure 27. Light-triggered release of indomethacin or naproxen reduced LPS-induced inflammatory responses of RAW 264.7 cells. (A) The NO content and (B) relative cell viability of RAW 264.7 cells treated with LPS and Os/BI NPs with/without light irradiation. (C) The NO content and (D) relative cell viability of RAW 264.7 cells treated with LPS and Os/BN NPs with/without light irradiation. $^{**}p < 0.01$, $^{***}p < 0.001$ were measured by Student's two-sided *t*-test. Data were presented as mean ± SD, $n = 3$ independent experiments.

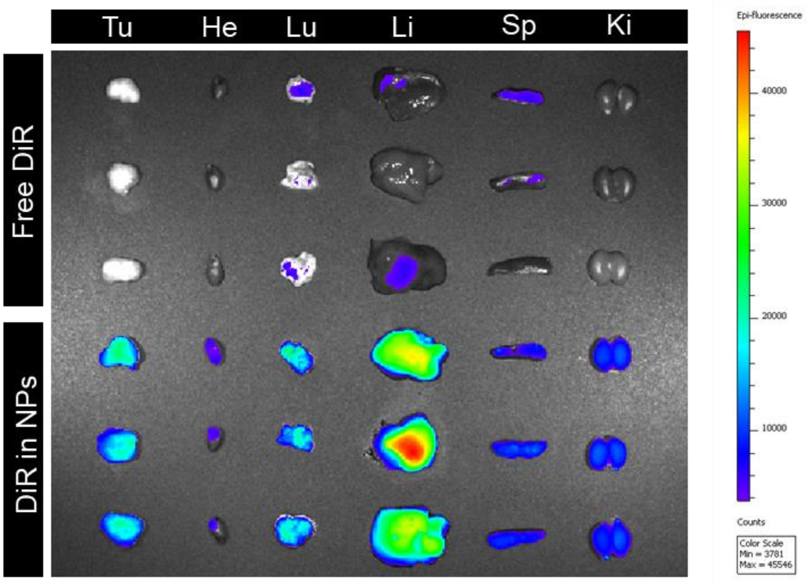

Supplementary Figure 28. Representative fluorescent images of the tumor and major organs 24 h after the injections of free DiR and DiR-labelled NPs.

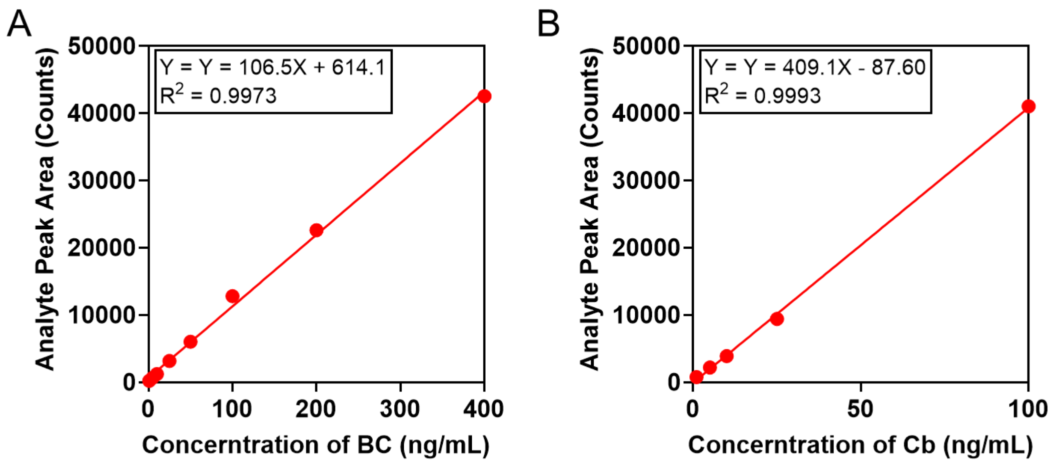

Supplementary Figure 29. Linear fitting of the concentrations of (A) BC and (B) Cb and analyte peak areas measured by LC/MS/MS system.

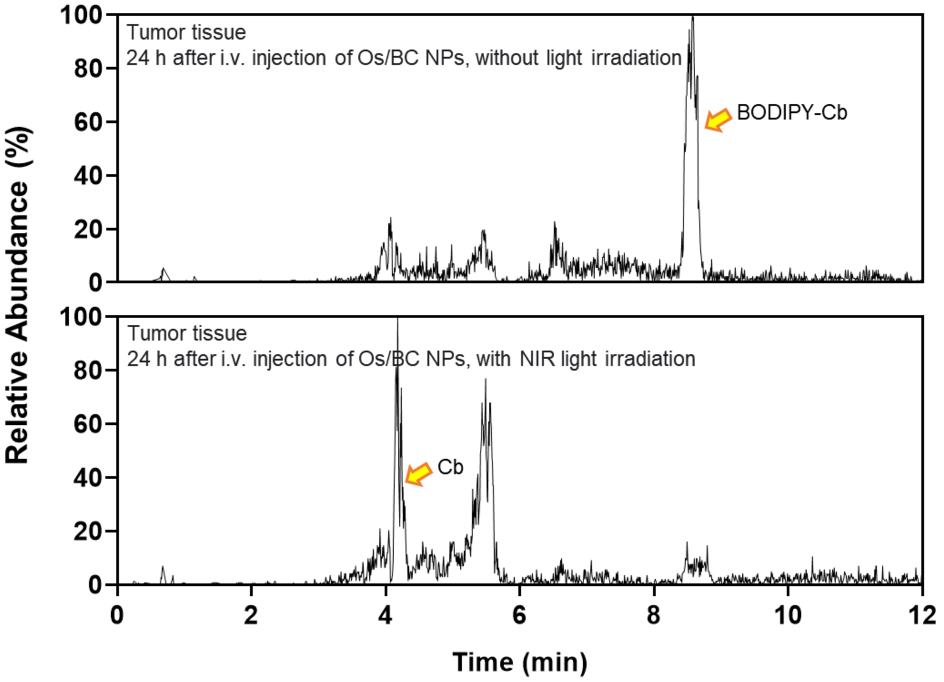

Supplementary Figure 30. Representative MRM chromatograms of tumor tissues 24 h post-treatments (i.v. injection of Os/BC NPs, with/without NIR light irradiation at tumors; light irradiation: 690 nm, 300 mW/cm^2, 10 min).

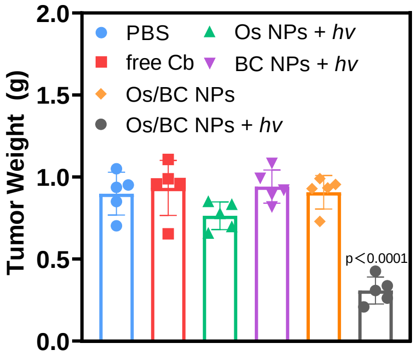

Supplementary Figure 31. Tumor weights at the end of the *in vivo* test (Day 13). Data were presented as mean ± SD, n = 5 mice. Statistic difference was measured by Student’s two-sided t-test.

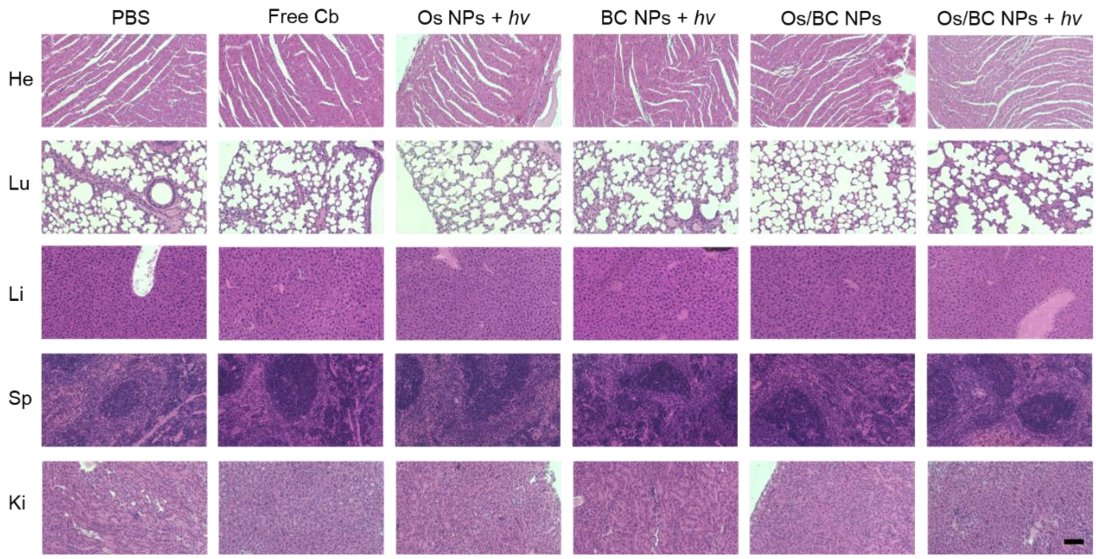

Supplementary Figure 32. H&E staining of major organs sections of the mice after different treatments. Scare bar: 200 μm.

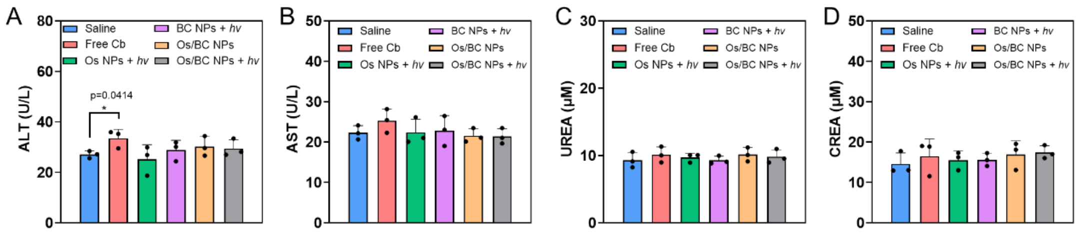

Supplementary Figure 33. Serum biochemistry (A. aminotransferase (ALT); B. aspartate aminotransferase (AST); C. urea and D. creatinine (CREA)) of the mice treated with different formulations twice within 14 days. Data were presented as mean ± SD, n = 3 independent tests. Statistic difference was measured by Student's two-sided t-test, $^{*}p < 0.05$.

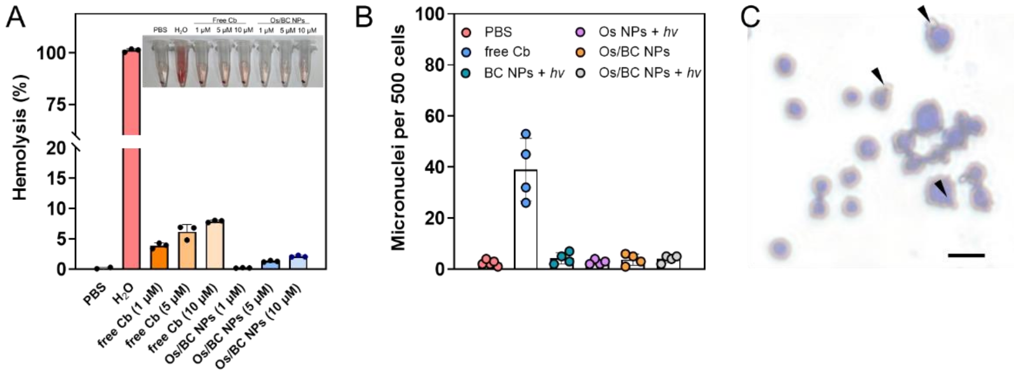

Supplementary Figure 34. (A) Hemolysis test of free Cb and Os/BC NPs at different concentrations. Data were presented as mean ± SD, *n* = 3 independent experiments. (B) Number of micronuclei in bone marrow cells after different treatments. Data were presented as mean ± SD, *n* = 4 mice. (C) Representative image of the bone marrow cells of the free Cb-treated mice. Black arrows indicate micronuclei. Scale bar: 10 μm.

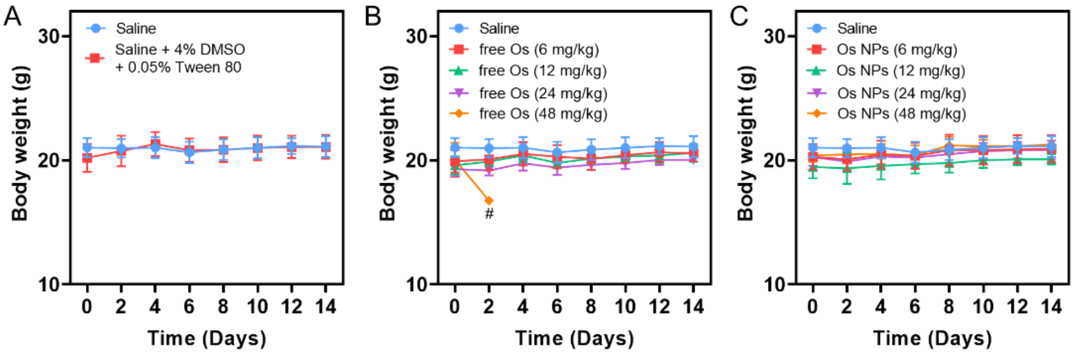

Supplementary Figure 35. The body weight of the mice treated with saline with/without DMSO and Tween 80 (A), free Os (B) and Os NPs (C) from 6 to 48 mg/kg (on basis of Os complex) by i.v. injection within 14 days (# euthanized due to weight loss). Data were presented as mean ± SD, *n* = 3 mice.

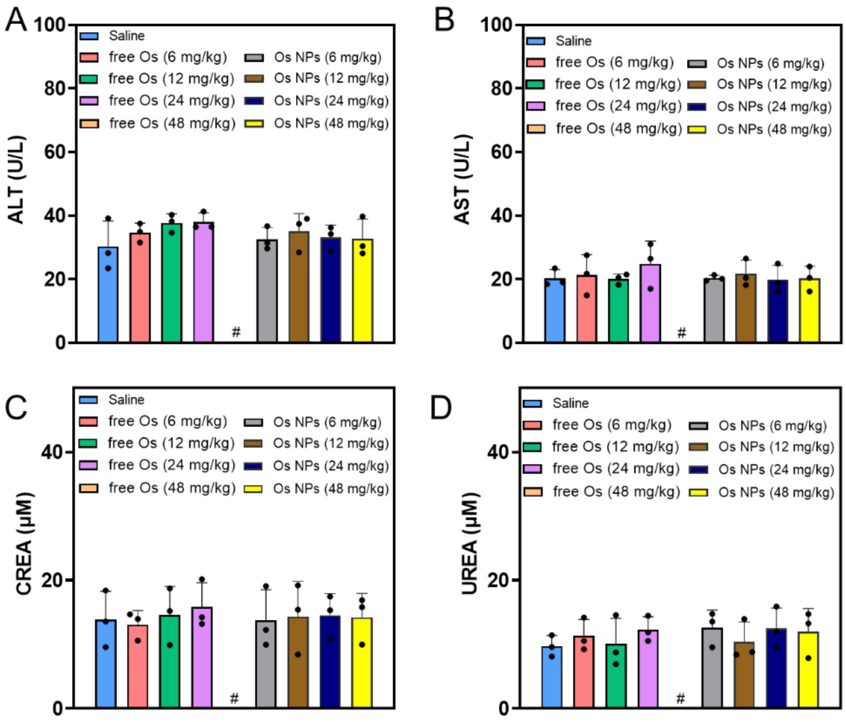

Supplementary Figure 36. Serum biochemistry (A. aminotransferase (ALT); B. aspartate aminotransferase (AST); C. urea and D. creatinine (CREA)) of the mice post-treated with free Os or Os NPs from 6 to 48 mg/kg by i.v. injection. (# euthanized due to weight loss). Data were presented as mean ± SD, n = 3 independent tests.

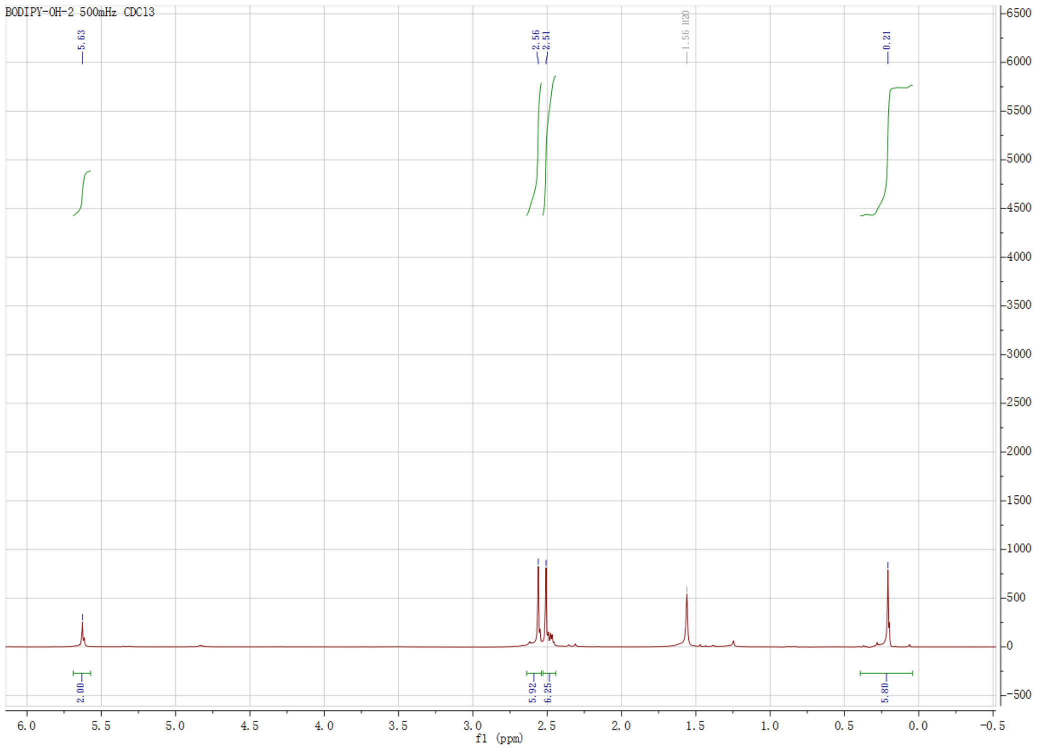

Supplementary Figure 37. ^{1}H-NMR spectrum of BODIPY photocage 2.

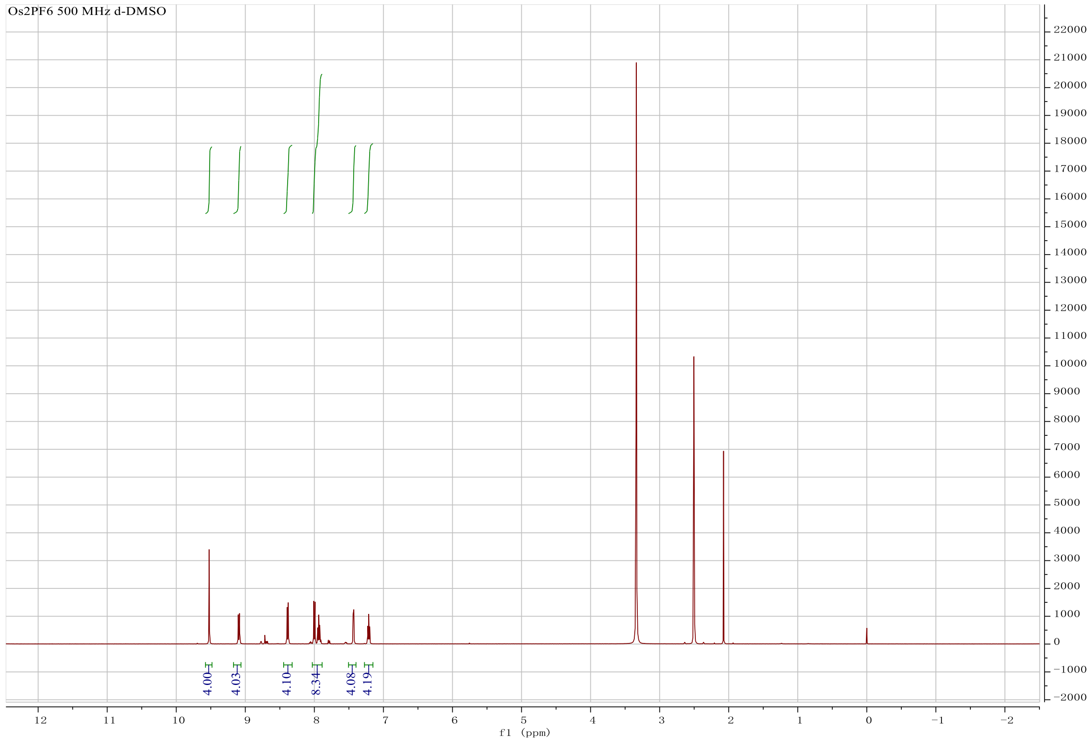

Supplementary Figure 38. ^{1}H-NMR spectrum of $Os(bptpy)_2{\cdot}2PF_6$.

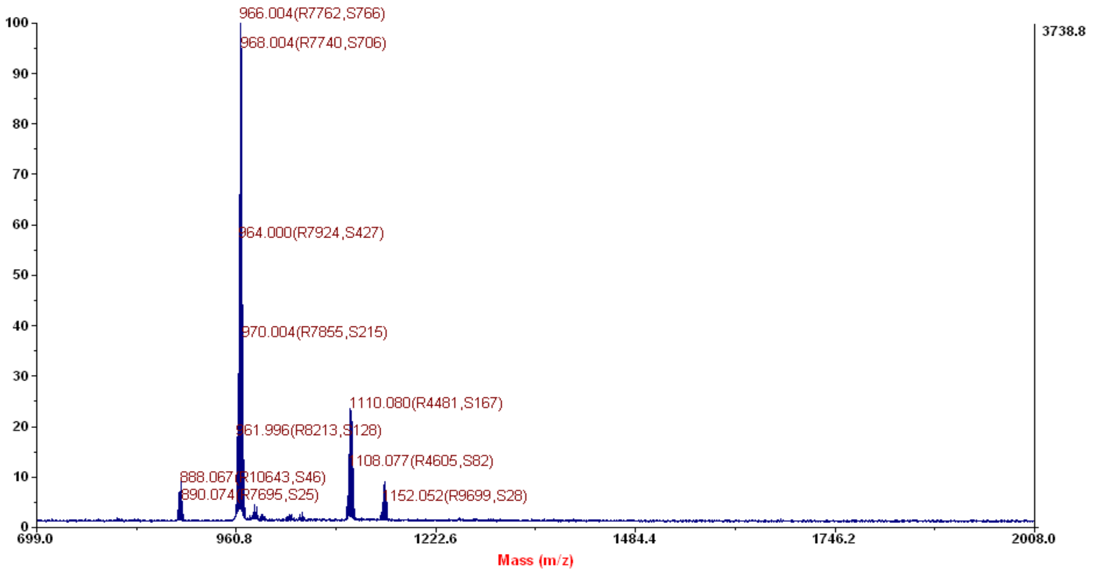

Supplementary Figure 39. MALDI-TOF MS spectrum of $Os(bptpy)_2 \cdot 2PF_6$.

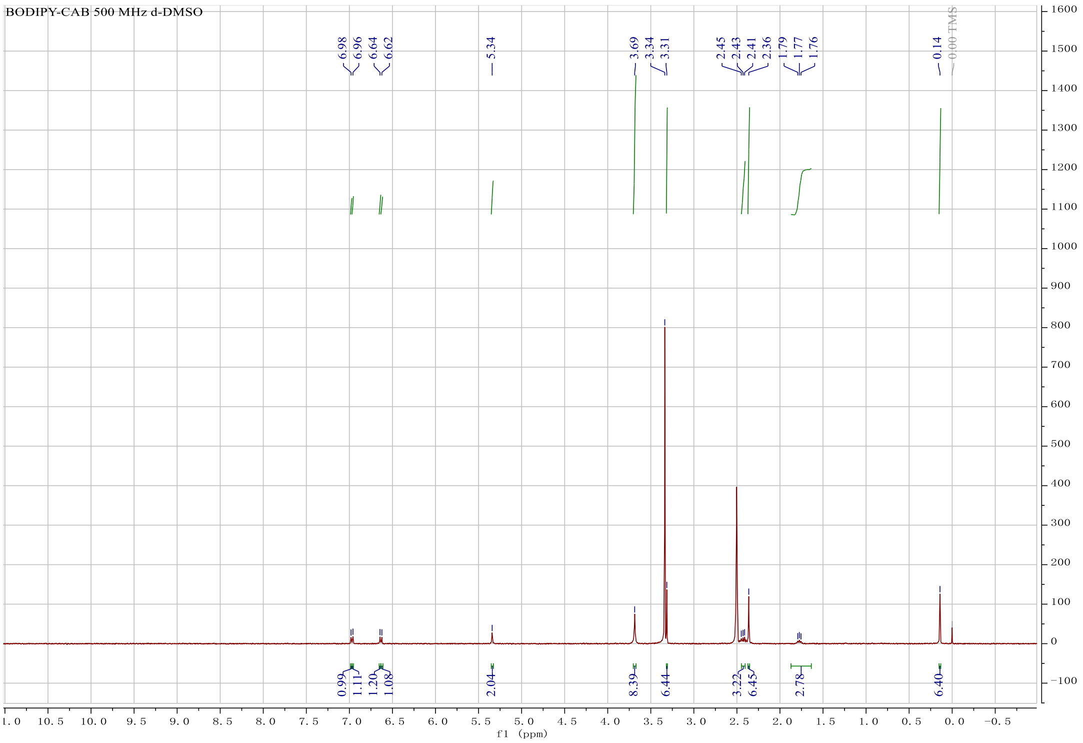

Supplementary Figure 40. ^{1}H-NMR spectrum of BODIPY-Cb.

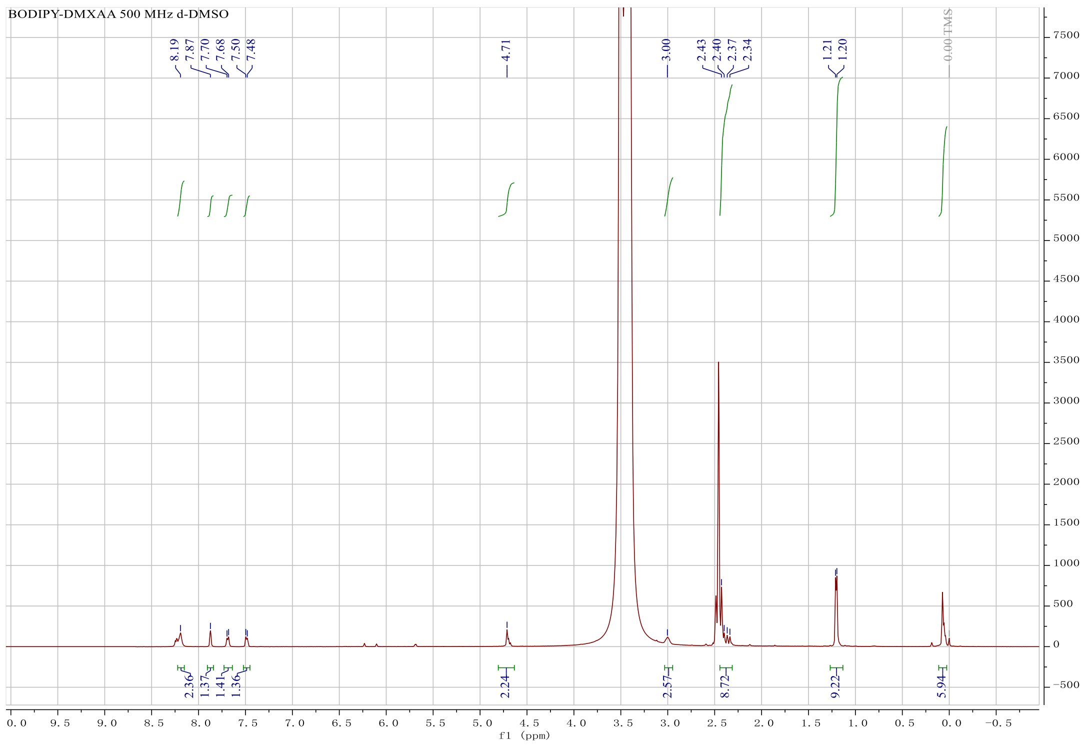

Supplementary Figure 41. ^{1}H-NMR spectrum of BODIPY-DMXAA.

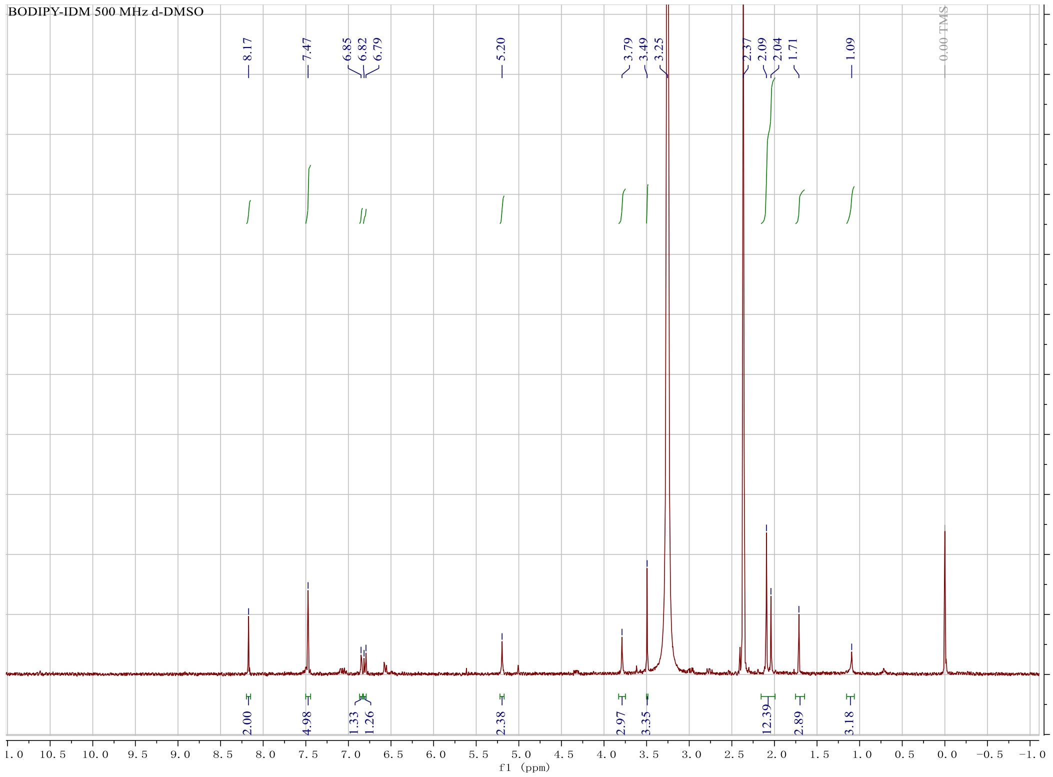

Supplementary Figure 42. ^{1}H-NMR spectrum of BODIPY-IDM.

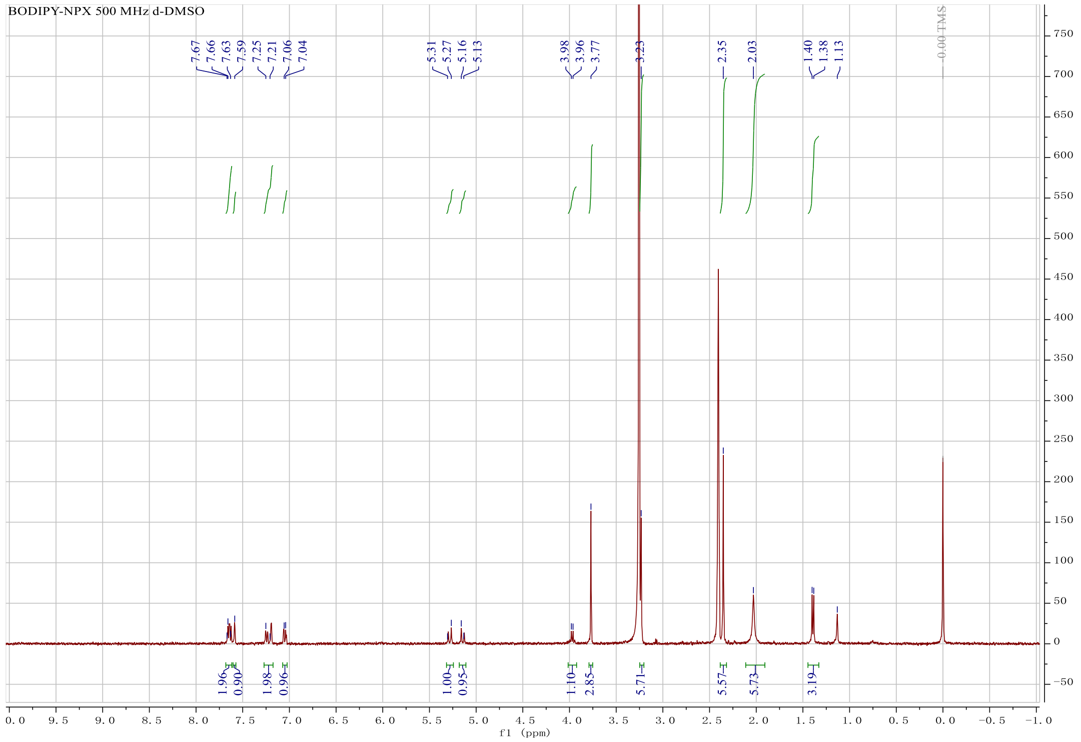

Supplementary Figure 43. ^{1}H-NMR spectrum of BODIPY-NPX.

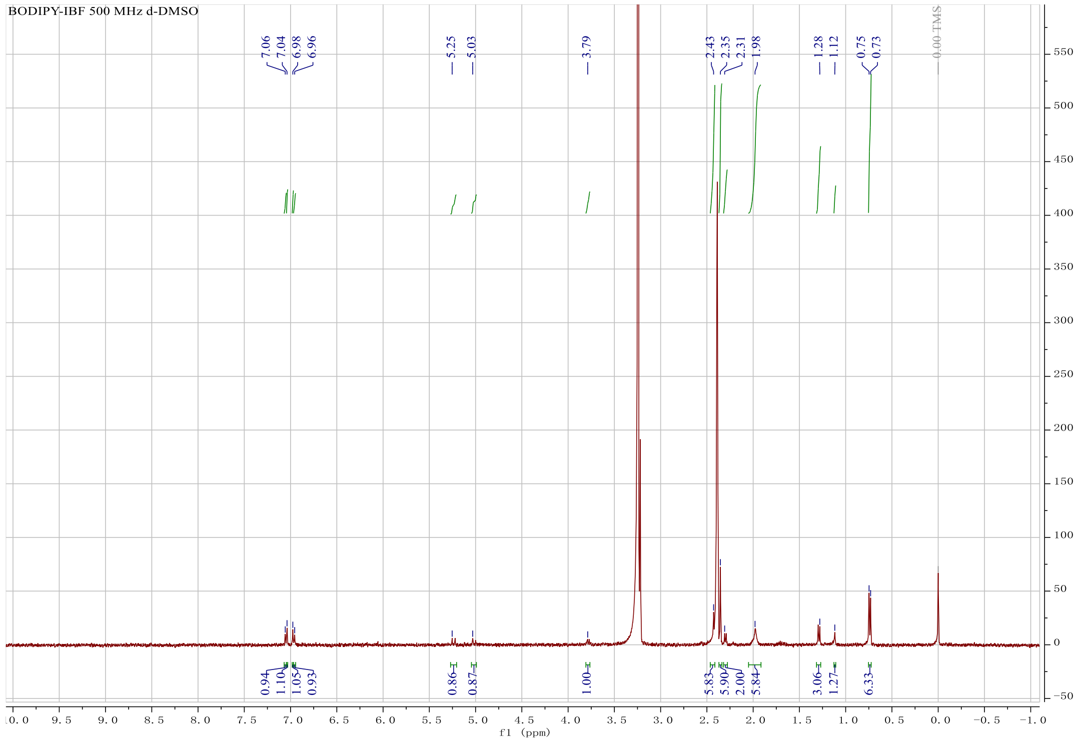

Supplementary Figure 44. ^{1}H-NMR spectrum of BODIPY-IBF.

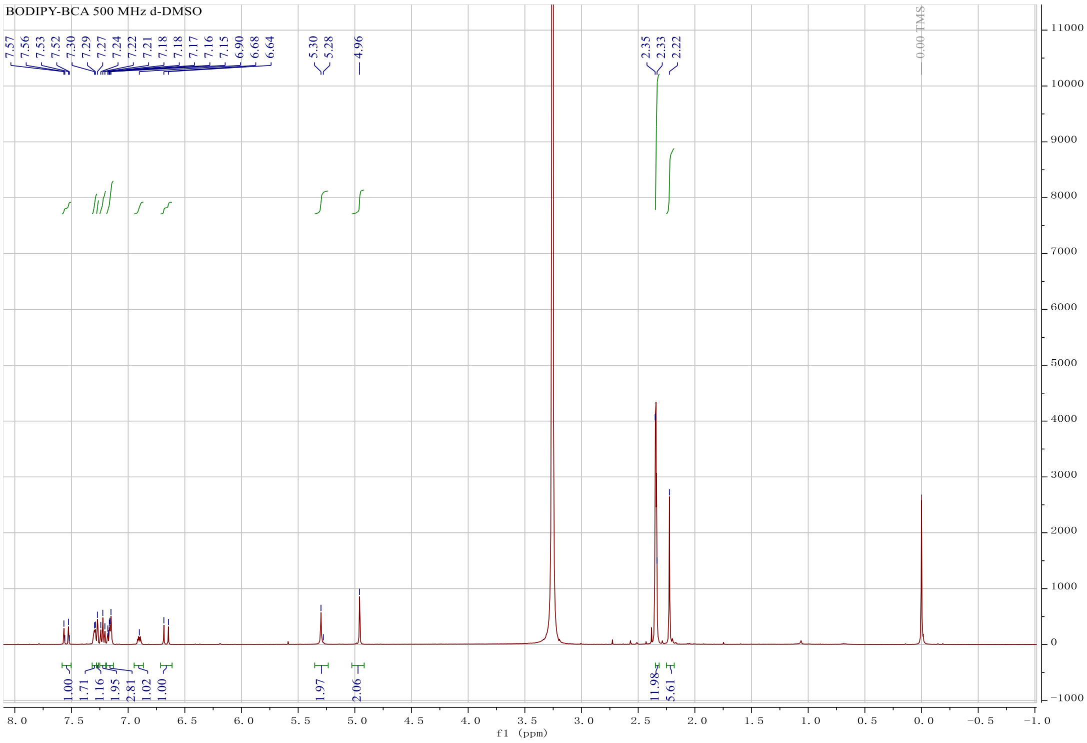

Supplementary Figure 45. ^{1}H-NMR spectrum of BODIPY-BCA.

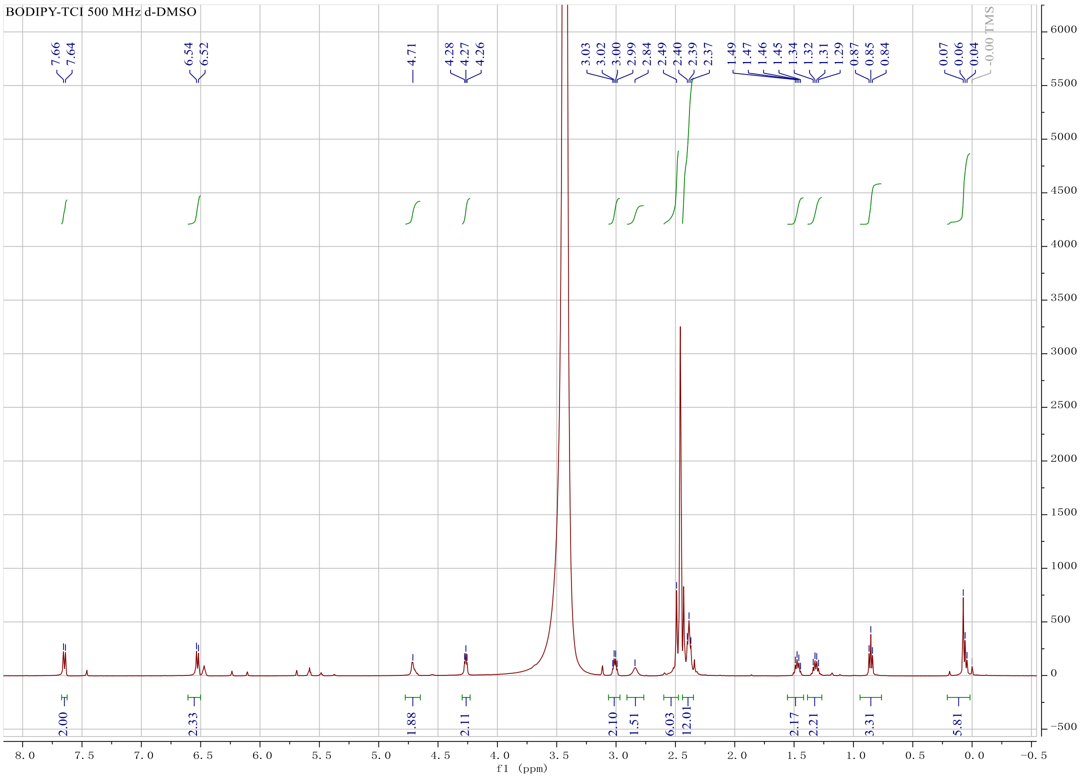

Supplementary Figure 46. ^{1}H-NMR spectrum of BODIPY-TCI.

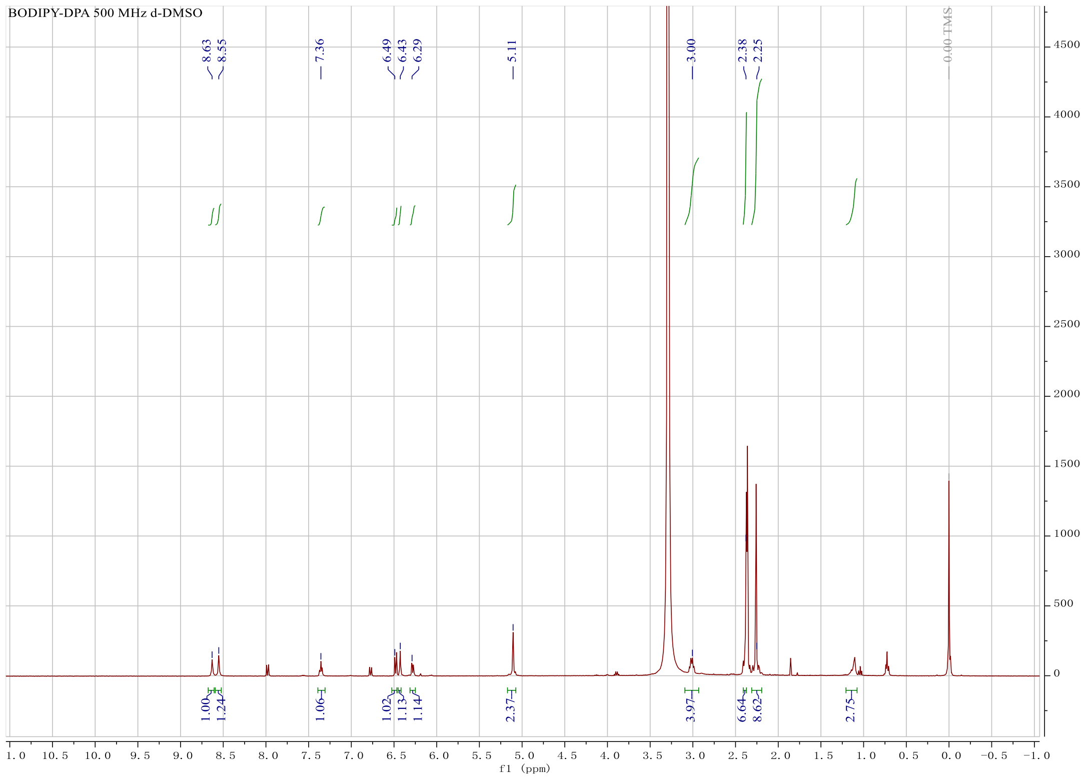

Supplementary Figure 47. ^{1}H-NMR spectrum of BODIPY-DPA.

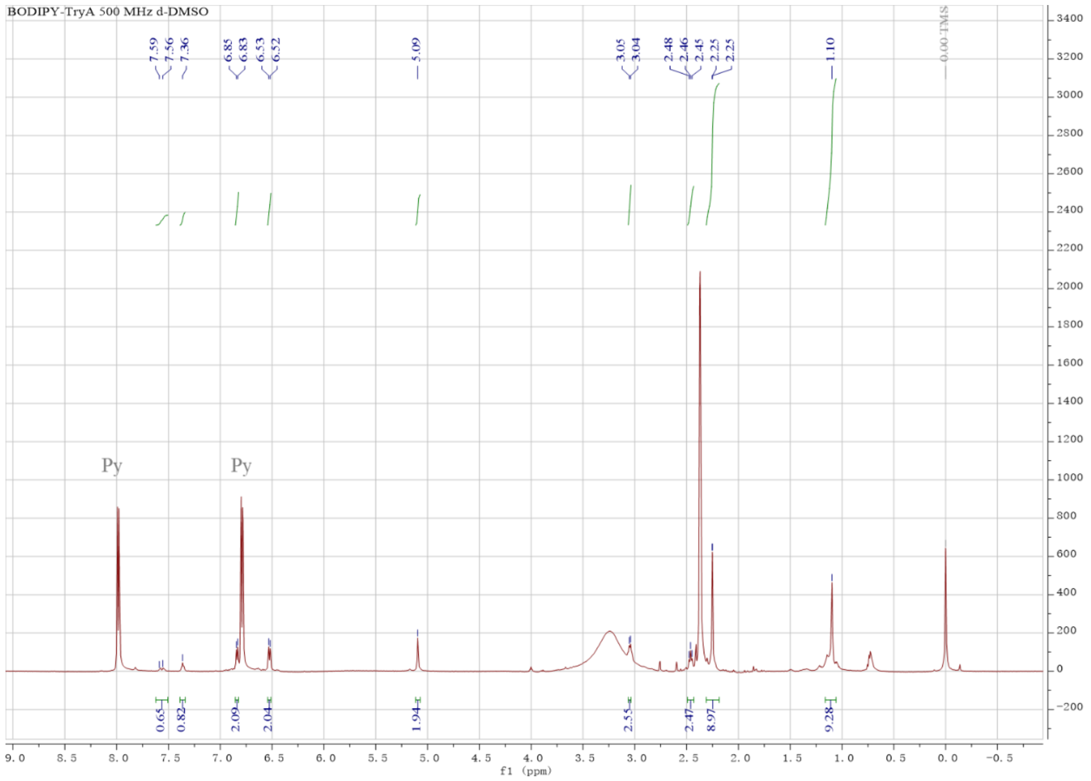

Supplementary Figure 48. ^{1}H-NMR spectrum of BODIPY-TyrA.

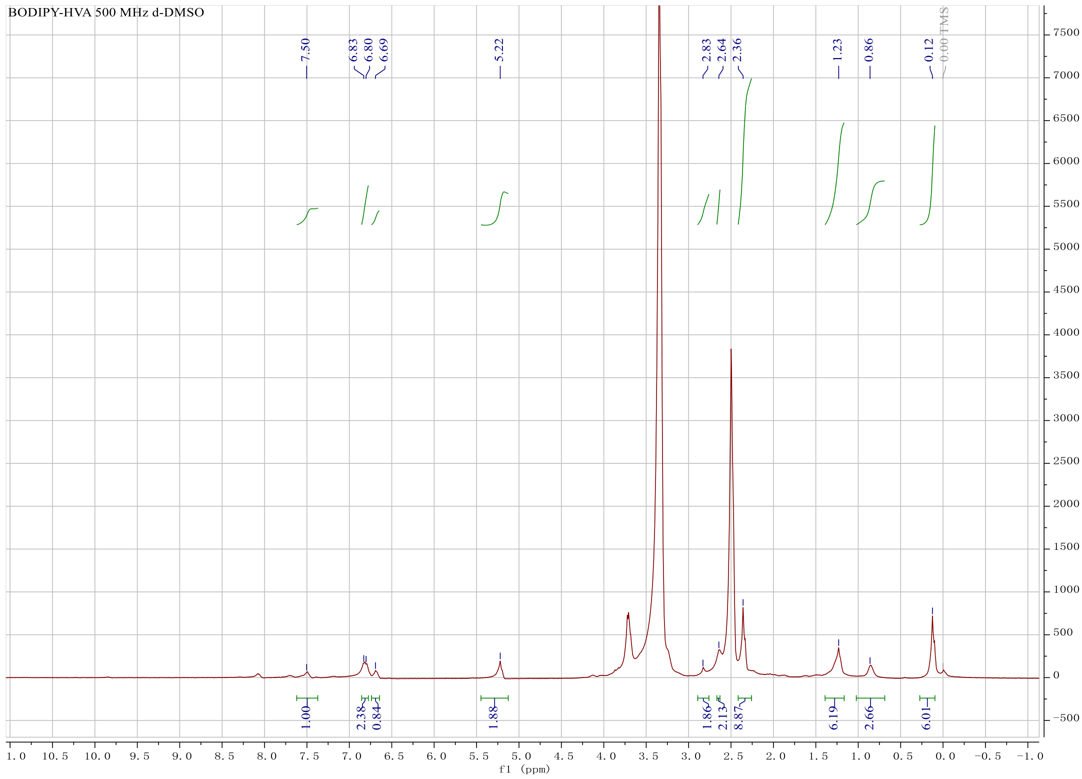

Supplementary Figure 49. ^{1}H-NMR spectrum of BODIPY-HVA.

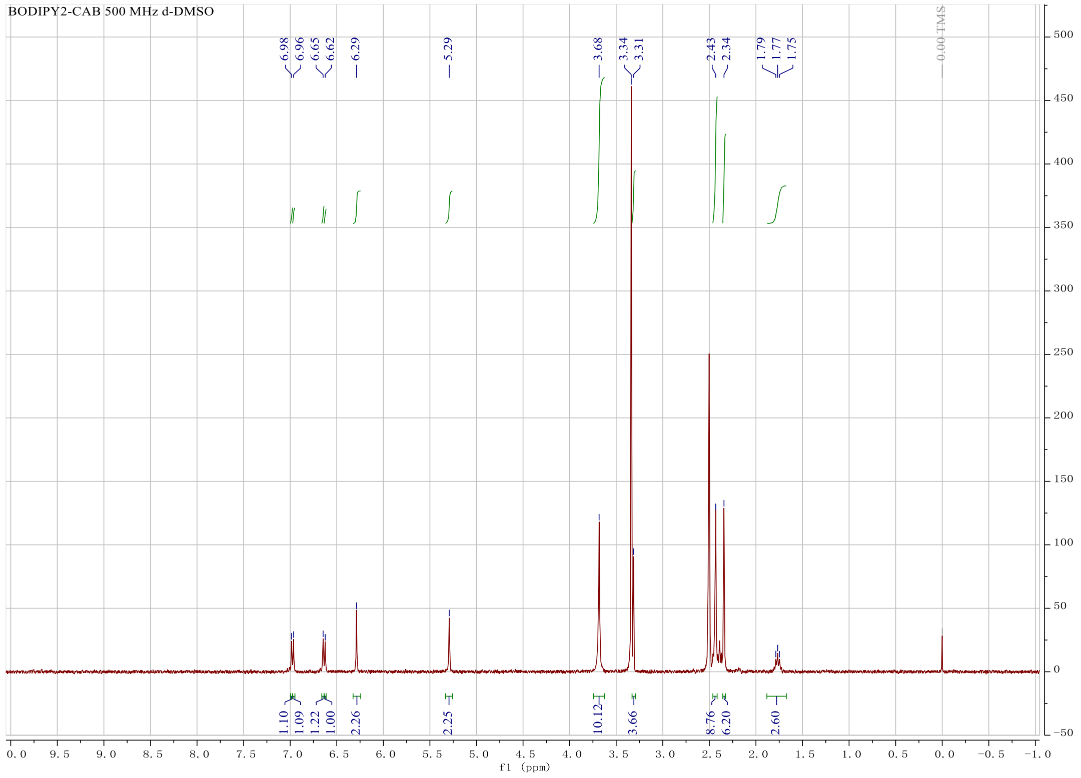

Supplementary Figure 50. ^{1}H-NMR spectrum of BODIPY2-Cb.

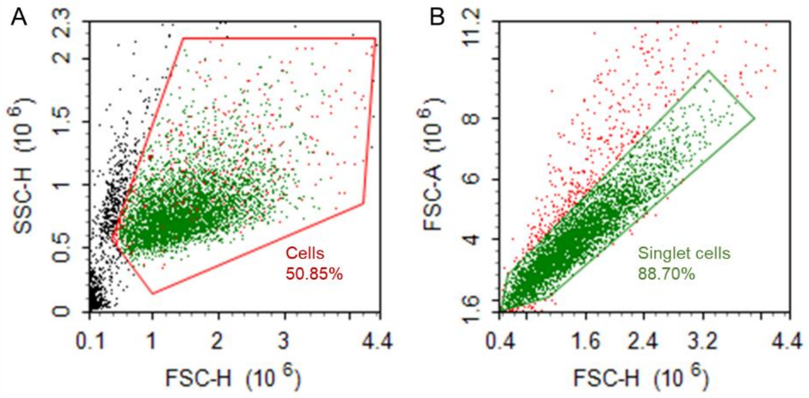

Supplementary Figure 51. Gating strategy for flow cytometry. (A) Representative gating strategy based on SSC-H and FSC-H parameters to select for cells. (B) Selection for singlet cells based on FSC-H/FSC-A. The gating strategy was used prior to flow cytometry analysis of cell apoptosis which showed in Figure 5e.

3. Supplementary Tables

Supplementary Table 1. Photophysical properties of BODIPY-Cb and $Os(bptpy)_2 \cdot 2PF_6$.

Compound	$\lambda_{abs, max}$ (nm)[a]	ε (M^{-1} cm^{-1})	$\lambda_{em, max}$ (nm)	T_1 lifetime (μs)	Energy level (eV)	
					S_1 state	T_1 state
BODIPY-Cb	543	8.94×10^4	570[b]	5.27[e]	2.19[g]	1.51[g]
$Os(bptpy)_2 \cdot 2PF_6$	491 (S_0-S_1)	7.35×10^4	498[c]	0.20[f]	2.23[g]	1.69[h]
	688 (S_0-T_1)	2.66×10^4	734[d]			

[a]In dichloromethane solution with 2% acetone, 10 μM;

[b]λ_{ex} = 543 nm; [c]λ_{ex} = 491 nm; [d]λ_{ex} = 688 nm;

[e]Calculated by decay trace of transient absorbance at 540 nm;

[f]Referred to previous study;[3]

[g]Calculated by TD-DFT;

[h]Calculated maximum T_1 emission wavelength (λ_{em}), $E = 1240/\lambda_{em, T1}$;

Supplementary Table 2. HPLC method for photolysis study.

Time (min)	Acetonitrile (0.1 % TFA, v/v)	H_2O (0.1 % TFA, v/v)
0	20%	80%
10	40%	60%
15	80%	20%
18	100%	0%
30	100%	0%

Supplementary Table 3. Quantum yields and cross sections of BODIPY-Cb prodrug in different conditions.

Compound	λ_{ex} (nm)	Conditions	Quantum yield of prodrug photolysis Φ_p (%)	Cross section $\Phi_p\varepsilon(\lambda_{ex})$ ($M^{-1}cm^{-1}$)	Quantum yield of drug release Φ_r(%)	Cross section $\Phi_r\varepsilon(\lambda_{ex})$ ($M^{-1}cm^{-1}$)
BODIPY-Cb	530	N_2	23.89 ± 3.05	16017.11 ± 2044.88	6.73 ± 2.12	6016.62 ± 1895.28
		Air	3.42 ± 1.09	2292.95 ± 730.79	$(2.56 \pm 0.37) \times 10^{-1}$	171.63 ± 24.81
	690	0.1 eq Os, N_2	0.84 ± 0.01	222.80 ± 2.90	0.73 ± 0.04	193.85 ± 9.70
		0.1 eq Os, Air	$(7.03 \pm 4.18) \times 10^{-3}$	1.87 ± 1.11	$(1.86 \pm 0.4) \times 10^{-3}$	0.49 ± 0.11

Supplementary Table 4. Quantum yields and cross sections of BODIPY prodrugs photolysis under NIR light.

Compound	λ_{ex} (nm)	Conditions	Quantum yield of photolysis Φ_p (%)	Cross section $\Phi_p\varepsilon(\lambda_{ex})^a$ (M^{-1} cm^{-1})	Quantum yield of drug release Φ_r(%)	Cross section $\Phi_r\varepsilon(\lambda_{ex})^a$ (M^{-1} cm^{-1})
BODIPY-Cb	690	0.1 eq Os, N_2	0.84 ± 0.01	222.80 ± 2.90	0.73 ± 0.04	193.85 ± 9.70
BODIPY-DMXAA	690	0.1 eq Os, N_2	0.87 ± 0.01	230.30 ± 1.43	0.71 ± 0.06	190.05 ± 16.63
BODIPY-IDM	690	0.1 eq Os, N_2	0.86 ± 0.02	229.15 ± 5.23	0.60 ± 0.04	158.79 ± 10.80
BODIPY-NPX	690	0.1 eq Os, N_2	0.85 ± 0.02	227.15 ± 4.42	0.73 ± 0.05	194.19 ± 13.33
BODIPY-IBF	690	0.1 eq Os, N_2	0.85 ± 0.04	226.34 ± 10.96	0.42 ± 0.04	111.67 ± 10.81
BODIPY-BCA	690	0.1 eq Os, N_2	0.80 ± 0.02	213.33 ± 7.55	0.75 ± 0.03	200.64 ± 7.30
BODIPY-TCI	690	0.1 eq Os, N_2	0.81 ± 0.06	215.84 ± 15.71	0.36 ± 0.04	95.21 ± 10.62
BODIPY-DPA	690	0.1 eq Os, N_2	0.86 ± 0.01	228.90 ± 3.59	0.60 ± 0.02	159.00 ± 4.58
BODIPY-TyrA	690	0.1 eq Os, N_2	0.84 ± 0.02	223.12 ± 6.28	0.40 ± 0.02	107.25 ± 5.46
BODIPY-HVA	690	0.1 eq Os, N_2	0.85 ± 0.04	224.98 ± 9.83	0.64 ± 0.03	170.82 ± 9.14

$^a\varepsilon(\lambda_{ex})$ here was the absorption coefficient of $Os(bptpy)_2 \cdot 2PF_6$ at 688 nm (ε = 26600 M^{-1} cm^{-1}).

Supplementary Table 5. Composition of Os/BC NP.

	Feeding weight (mg)	Weight after purification (mg)	Encapsulation Efficiency	Loading Capacity
PLA5k-mPEG5k	400	/	/	/
$Os(bptpy)_2 \cdot 2PF_6$	3.02	0.89	29.43%	0.22%
BODIPY-Cb	1.94	1.11	55.79%	0.27%

Supplementary Table 6. Size and PDI of Os NPs, BC NPs and Os/BC NPs.

	Size (nm)	PDI
Blank NPs	47.20 ± 0.35	0.10 ± 0.01
Os NPs	54.58 ± 0.60	0.22 ± 0.02
BC NPs	56.20 ± 2.28	0.16 ± 0.03
Os/BC NPs	53.51 ± 0.70	0.13 ± 0.02

Supplementary References

1. Lv, W.; Li, Y.; Li, F.; Lan, X.; Zhang, Y.; Du, L.; Zhao, Q.; Phillips, D. L.; Wang, W., Upconversion-like photolysis of BODIPY-based prodrugs via a one-photon process. *J. Am. Chem. Soc.* **2019,** *141* (44), 17482-17486.
2. Sasaki, Y.; Oshikawa, M.; Bharmoria, P.; Kouno, H.; Hayashi-Takagi, A.; Sato, M.; Ajioka, I.; Yanai, N.; Kimizuka, N., Near‐infrared optogenetic genome engineering based on photon-upconversion hydrogels. *Angew. Chem. Int. Ed.* **2019,** *58* (49), 17827-17833.
3. Sasaki, Y.; Amemori, S.; Kouno, H.; Yanai, N.; Kimizuka, N., Near infrared-to-blue photon upconversion by exploiting direct S–T absorption of a molecular sensitizer. *Journal of Materials Chemistry C* **2017,** *5* (21), 5063-5067.
