# Supplementary material for: Near-infrared light-triggered prodrug photolysis by one-step energy transfer
Source: Nat Commun. 2023 Dec 7;14:8112. doi: 10.1038/s41467-023-43805-y (PMC10703928; doi:10.1038/s41467-023-43805-y)
Supplement: Supplementary file 3 — Reporting Summary [file 41467_2023_43805_MOESM3_ESM.pdf]

nature portfolio

Corresponding author(s): Weiping Wang

Last updated by author(s): Nov 20, 2023

Reporting Summary

Nature Portfolio wishes to improve the reproducibility of the work that we publish. This form provides structure for consistency and transparency in reporting. For further information on Nature Portfolio policies, see our Editorial Policies and the Editorial Policy Checklist.

Statistics

For all statistical analyses, confirm that the following items are present in the figure legend, table legend, main text, or Methods section.

n/a	Confirmed	
☐	☒	The exact sample size (*n*) for each experimental group/condition, given as a discrete number and unit of measurement
☐	☒	A statement on whether measurements were taken from distinct samples or whether the same sample was measured repeatedly
☐	☒	The statistical test(s) used AND whether they are one- or two-sided<br>*Only common tests should be described solely by name; describe more complex techniques in the Methods section.*
☒	☐	A description of all covariates tested
☐	☒	A description of any assumptions or corrections, such as tests of normality and adjustment for multiple comparisons
☐	☒	A full description of the statistical parameters including central tendency (e.g. means) or other basic estimates (e.g. regression coefficient) AND variation (e.g. standard deviation) or associated estimates of uncertainty (e.g. confidence intervals)
☐	☒	For null hypothesis testing, the test statistic (e.g. *F*, *t*, *r*) with confidence intervals, effect sizes, degrees of freedom and *P* value noted<br>*Give P values as exact values whenever suitable.*
☒	☐	For Bayesian analysis, information on the choice of priors and Markov chain Monte Carlo settings
☒	☐	For hierarchical and complex designs, identification of the appropriate level for tests and full reporting of outcomes
☒	☐	Estimates of effect sizes (e.g. Cohen's *d*, Pearson's *r*), indicating how they were calculated

Our web collection on statistics for biologists contains articles on many of the points above.

Software and code

Policy information about availability of computer code

Data collection	ZEN 2.3 (blue edition) ; Living Image Software (4.5.5, IVIS Imaging Systems, perkinElmer); Novoexpress (1.6);
Data analysis	Graphpad Prism 8.0 was used for linear fitting and normalization of the spectrum data.<br>ZEN 2.3 was used to merge and analyze confocal images as described in the Methods section.<br>Living Image Software (4.5.5, IVIS Imaging Systems, perkinElmer) was used to analyze the in vivo images.<br>Novoexpress (1.6) was used to analyze the flowcytometry data, including gating and measuring cell amounts.<br>All DFT and TD-DFT calculations were carried out in the Gaussian 16C.01 package.

For manuscripts utilizing custom algorithms or software that are central to the research but not yet described in published literature, software must be made available to editors and reviewers. We strongly encourage code deposition in a community repository (e.g. GitHub). See the Nature Portfolio guidelines for submitting code & software for further information.

Data

Policy information about availability of data

All manuscripts must include a data availability statement. This statement should provide the following information, where applicable:

- Accession codes, unique identifiers, or web links for publicly available datasets
- A description of any restrictions on data availability
- For clinical datasets or third party data, please ensure that the statement adheres to our policy

Data generated or analyzed in this study are all included in the article and Supplementary Information file. The full image dataset is available from the corresponding author upon request. Source data are provided with this paper.

Research involving human participants, their data, or biological material

Policy information about studies with human participants or human data. See also policy information about sex, gender (identity/presentation), and sexual orientation and race, ethnicity and racism.

Reporting on sex and gender	N/A
Reporting on race, ethnicity, or other socially relevant groupings	N/A
Population characteristics	N/A
Recruitment	N/A
Ethics oversight	N/A

Note that full information on the approval of the study protocol must also be provided in the manuscript.

Field-specific reporting

Please select the one below that is the best fit for your research. If you are not sure, read the appropriate sections before making your selection.

☒ Life sciences ☐ Behavioural & social sciences ☐ Ecological, evolutionary & environmental sciences

For a reference copy of the document with all sections, see nature.com/documents/nr-reporting-summary-flat.pdf

Life sciences study design

All studies must disclose on these points even when the disclosure is negative.

Sample size	Statistical methods were not used for predetermining sample sizes. Sample size was determined based on our experiences of similar experiments conducted by our group (J Am Chem Soc, 2019, 141, 44, 17482-17486; J Nanobiotechnology, 2021, 19, 357). All the sample size included at least 3 independent experiments, and the exact number was described in the legends of Figures.
Data exclusions	No data were excluded from analysis.
Replication	At least three independent studies were carried out. All of the replicates showed similar results.
Randomization	Randomization was not relevant to the study. No population studies were involved, and the study methods are inherently unbiased and do not require further randomization. In cell experiments, replicate experiments were performed using different batches. Cells or animals with different treatments l were analyzed equally with no sub-sampling.
Blinding	Blinding was not relevant to all the chemical experiments and cell tests, which were conducted through standard protocols and procedures. Data were collected and counted objectively, which does not bias any particular result due to knowledge of sample identity.<br>In animal tests, histological study were kindly performed in blinded fashion by a pathologist from the Department of Pathology, The University of Hong Kong.

Reporting for specific materials, systems and methods

We require information from authors about some types of materials, experimental systems and methods used in many studies. Here, indicate whether each material, system or method listed is relevant to your study. If you are not sure if a list item applies to your research, read the appropriate section before selecting a response.

Materials & experimental systems

n/a	Involved in the study	
x		Antibodies
	x	Eukaryotic cell lines
x		Palaeontology and archaeology
	x	Animals and other organisms
x		Clinical data
x		Dual use research of concern
x		Plants

Methods

n/a	Involved in the study	
x		ChIP-seq
	x	Flow cytometry
x		MRI-based neuroimaging

Eukaryotic cell lines

Policy information about cell lines and Sex and Gender in Research

Cell line source(s)	Human Cervical Adenocarcinoma cell (HeLa) were purchased from the Cell Bank of Chinese Academy of Sciences (China).
Authentication	Cell lines were frequently checked according to their morphological features and no further authentication was performed.
Mycoplasma contamination	Cell lines were not tested for mycoplasma contamination but no indication of contamination was observed.
Commonly misidentified lines (See ICLAC register)	No commonly misidentified cell lines were used.

Animals and other research organisms

Policy information about studies involving animals; ARRIVE guidelines recommended for reporting animal research, and Sex and Gender in Research

Laboratory animals	BALB/c nude mice (age 4 weeks, about 20 g) were used for tumor implantation and further study. All mice were obtained from the Experimental Animal Center of University with access to food and water ad libitum and maintained under pathogen-free condition. Other environmental conditions were: photo-period control with 12-h light/12-h dark cycle; temperatures of 16-26°C with 30-70% humidity; 100% fresh air supply with 15 air changes per hour.
Wild animals	No wild animals were used in this study.
Reporting on sex	Female mice were used since we used the cervical tumor model.
Field-collected samples	No field-collected samples were used in this study
Ethics oversight	The animal experiment and procedures were approved by the Committee on the Use of Live Animals in Teaching & Research (CULATR), The University of Hong Kong. (Protocol No. 4381-17).

Note that full information on the approval of the study protocol must also be provided in the manuscript.

Plants

Seed stocks	N/A
Novel plant genotypes	N/A
Authentication	N/A

Flow Cytometry

Plots

Confirm that:

☒ The axis labels state the marker and fluorochrome used (e.g. CD4-FITC).

☒ The axis scales are clearly visible. Include numbers along axes only for bottom left plot of group (a 'group' is an analysis of identical markers).

☒ All plots are contour plots with outliers or pseudocolor plots.

☒ A numerical value for number of cells or percentage (with statistics) is provided.

Methodology

Sample preparation	The cells stained with fluorescent probes were collected by trypsin (0.25% with EDTA) after 3-min incubation and centrifugation (300 g, 3 min). The cells were washed by PBS for 3 times before measurements.
Instrument	Agilent NovoCyte Quanteon analyzer
Software	NovoExpress Software (1.6)
Cell population abundance	All the displayed cell population (after gating) are larger than 10000 to ensure the cell population abundance.
Gating strategy	Gating was based on FSC/SSC of the cancer cells. All the single-cell population was used for analysis.

☒ Tick this box to confirm that a figure exemplifying the gating strategy is provided in the Supplementary Information.
